# Lipoplex‐Functionalized Thin‐Film Surface Coating Based on Extracellular Matrix Components as Local Gene Delivery System to Control Osteogenic Stem Cell Differentiation

**DOI:** 10.1002/adhm.202201978

**Published:** 2022-11-29

**Authors:** Catharina Husteden, Yazmin A. Brito Barrera, Sophia Tegtmeyer, João Borges, Julia Giselbrecht, Matthias Menzel, Andreas Langner, João F. Mano, Christian E. H. Schmelzer, Christian Wölk, Thomas Groth

**Affiliations:** ^1^ Institute of Pharmacy Department of Medicinal Chemistry Martin Luther University Halle‐Wittenberg Wolfgang‐Langenbeck‐Str. 4 06120 Halle (Saale) Germany; ^2^ Institute of Pharmacy Department of Biomedical Materials Martin Luther University Halle‐Wittenberg Heinrich‐Damerow‐Str. 4 06120 Halle (Saale) Germany; ^3^ Department of Chemistry CICECO – Aveiro Institute of Materials University of Aveiro Campus Universitário de Santiago Aveiro 3810‐193 Portugal; ^4^ Department of Biological and Macromolecular Materials Fraunhofer Institute for Microstructure of Materials and Systems (IMWS) Walter‐Hülse‐Str. 1 06120 Halle (Saale) Germany; ^5^ Institute of Pharmacy Pharmaceutical Technology Faculty of Medicine Leipzig University 04317 Leipzig Germany; ^6^ Interdisciplinary Center of Materials Science Martin Luther University Halle‐Wittenberg Heinrich‐Damerow‐Str. 4 06120 Halle (Saale) Germany

**Keywords:** bone morphogenic protein 2, chondroitin sulfate, collagen I, human adipose‐derived mesenchymal stem cells, lipoplexes, osteogenic differentiation, polyelectrolyte multilayers

## Abstract

A gene‐activated surface coating is presented as a strategy to design smart biomaterials for bone tissue engineering. The thin‐film coating is based on polyelectrolyte multilayers composed of collagen I and chondroitin sulfate, two main biopolymers of the bone extracellular matrix, which are fabricated by layer‐by‐layer assembly. For further functionalization, DNA/lipid‐nanoparticles (lipoplexes) are incorporated into the multilayers. The polyelectrolyte multilayer fabrication and lipoplex deposition are analyzed by surface sensitive analytical methods that demonstrate successful thin‐film formation, fibrillar structuring of collagen, and homogenous embedding of lipoplexes. Culture of mesenchymal stem cells on the lipoplex functionalized multilayer results in excellent attachment and growth of them, and also, their ability to take up cargo like fluorescence‐labelled DNA from lipoplexes. The functionalization of the multilayer with lipoplexes encapsulating DNA encoding for transient expression of bone morphogenetic protein 2 induces osteogenic differentiation of mesenchymal stem cells, which is shown by mRNA quantification for osteogenic genes and histochemical staining. In summary, the novel gene‐functionalized and extracellular matrix mimicking multilayer composed of collagen I, chondroitin sulfate, and lipoplexes, represents a smart surface functionalization that holds great promise for tissue engineering constructs and implant coatings to promote regeneration of bone and other tissues.

## Graphical abstract

An extracellular matrix‐inspired polyelectrolyte multilayer film is functionalized with lipoplexes and used as gene activated biomaterial surface coating for application in regenerative medicine. Collagen I‐chondroitin sulfate multilayers with embedded lipoplexes having plasmids encoding for bone morphogenic protein 2 gene promote adhesion and growth of mesenchymal stem cells and transfect them efficiently inducing their osteogenic differentiation.

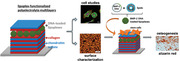

## 1 Introduction

Over the past few decades, the development of smart multifunctional biomaterials with the ability to control the behavior of stem cells on demand has become a powerful strategy in regenerative medicine and cell therapies.^[^ ^1^, ^2^ ^]^ For instance, such stem cell‐based therapies bear new chances to regenerate critical size bone defects from severe fractures or bone tissue loss after surgery. The osteogenic differentiation of mesenchymal stem cells is important for the healing of bone fractures and osteogenic diseases such as disorders of bone metabolism (osteoporosis).^[^ ^3^, ^4^, ^5^, ^6^ ^]^ Various studies have determined characteristics and modifications of biomaterials that enable initiation of stem cell osteogenesis and represent promising approaches for clinical use. These approaches include materials that can mimic the bone microenvironment,^[^ ^7^, ^8^ ^]^ materials with specific mechanical properties which stimulate bone tissue formation,^[^ ^9^ ^]^ and materials which can release or control the activity of osteoinductive growth factors.^[^ ^10^, ^11^, ^12^ ^]^

A straightforward strategy for the functionalization of biomaterials involves emulating the properties of the extracellular matrix (ECM) for the formation of an artificial microenvironment that enables a precise control of cell behavior and function.^[^ ^13^ ^]^ Due to interaction of ECM components with cell surface receptors such as integrins, ECM regulates cell proliferation, migration, and differentiation.^[^ ^14^, ^15^ ^]^ Indeed, the ECM is a highly versatile and dynamic compartment that can support development, function, and regeneration of tissues and organs by modulating the production, degradation, and remodeling of its components.^[^ ^16^ ^]^ Therefore, the development of surface coatings mimicking the native ECM structure and function is of considerable interest to functionalize implant materials. In this context, a simple and versatile method that can effectively immobilize bio‐functional molecules onto various materials and surfaces, with dynamic control of the surface topological and mechanical properties, is of upmost interest. The layer‐by‐layer (LbL) technology, well‐known from the pioneering work by Decher et al. on the development of polyelectrolyte multilayers (PEMs) on solid surfaces by alternating deposition of oppositely charged polyelectrolytes, has evolved into a very simple and cost effective yet highly versatile and efficient surface modification and functionalization technology. LbL technique allows the production of multifunctional thin film coatings with precise control of the film composition, structure, properties, and functions at the nanoscale.^[^ ^17^, ^18^ ^]^ A further advantage of LbL is that it can be performed by different methods such as dip‐coating, spray coating, and spin‐coating protocols allowing the coating of different materials and designs also in a time‐saving manner.^[^ ^18^ ^]^ Indeed, PEMs have been broadly used as reservoir for either the surface immobilization or encapsulation of bioactive molecules, more precisely drugs and proteins, to engineer bio‐functional materials by choice of polyelectrolytes and complexation conditions for regenerative medicine strategies.^[^ ^19^, ^20^, ^21^, ^22^, ^23^ ^]^ Type I collagen (Col) and chondroitin sulfate (Cs) are components of the ECM of bone. Col is the main organic component of the bone ECM and a perfect material in tissue engineering because of its excellent biodegradability, biocompatibility, and cell‐attracting properties. In fact, Col has drawn much attention for biomaterial development due to the existence of binding sites for cell receptors, cytokines, and other ECM components.^[^ ^19^, ^24^, ^25^, ^26^ ^]^ Cs is involved in cell recognition, intracellular signaling, and on the interaction between ECM components and cell‐surface glycoproteins.^[^ ^27^ ^]^ As such, Cs can enhance bone regeneration; thus, being used for the functionalization of PEMs for improved mineral deposition and osteogenesis.^[^ ^28^ ^]^

The functionalization of biomaterials with tissue relevant growth factors is also a promising strategy in tissue engineering. In the field of bone tissue engineering, the bone morphogenetic protein‐2 (BMP‐2) is a promising cytokine. Several studies have demonstrated that the growth factor BMP‐2 can be applied to stimulate bone healing and improve osteogenesis/osteointegration.^[^ ^29^, ^30^, ^31^, ^32^, ^33^ ^]^ For example, recombinant BMP‐2 is applied in the clinic for treatment of non‐union bone injuries, open tibia fractures, and spinal fusion in FDA‐approved systems for bone regeneration.^[^ ^8^, ^34^ ^]^ However, due to some persistent issues, including the need of loading large amounts of the recombinant BMP‐2 into the biomaterial, a burst release of supra‐physiological concentrations of BMP‐2 as well as the risk of unregulated and ectopic bone formation in vivo, the current clinical utilization of BMP‐2 has limitations,^[^ ^35^ ^]^ which forces the development of micro‐ or nanostructured delivery systems for BMP‐2^[^ ^36^ ^]^ or novel gene‐activated matrices.^[^ ^37^ ^]^

To overcome the existing drawbacks of BMP‐2 functionalized biomaterials, spatially limited acting in situ transfection systems gained attention to ensure a local cytokine production mediated by transfected cells. Surface‐mediated transfection strategies are based on a concept in which viral or non‐viral vectors embedded in matrix materials can promote a local, physiological, and/or sustained expression of a gene encoding for a therapeutic protein.^[^ ^38^ ^]^ By immobilizing plasmid DNA (pDNA) on surfaces, such as implants, surface‐mediated gene delivery achieved remarkable transient cell transfection and therapeutic effects, both in vivo and in vitro.^[^ ^39^, ^40^ ^]^ Despite their potential for tissue engineering, the use of viral vectors for gene delivery is limited by a high risk of immunogenicity and a certain risk for carcinogenicity. Therefore, current research is increasingly focusing on non‐viral vectors.^[^ ^41^ ^]^ Promising new methods are studied to find non‐viral vectors to achieve comparable gene transfer efficiency to viral vector equivalents. New transfection systems such as polymers, lipids, nanoparticles, and physical methods are studied to reduce cost, and increase safety and transfection efficiency.^[^ ^42^, ^43^ ^]^ For example, Olden et al. used cationic polyplexes for gene delivery into primary human T cells.^[^ ^44^ ^]^ Non‐viral gene delivery approaches have been specifically explored in cell‐based therapies because of their desirable safety profiles and simplicity of the preparation process when compared to viral vectors.^[^ ^45^ ^]^ However, non‐viral vectors are not suitable for systemic application in bone regeneration because DNA complexes carry the risk of transfection of undesired cell types and systemic side effects in vivo.

Previous studies have consistently demonstrated that electrostatic‐driven LbL assembly is a powerful and simple technique to functionalize biomaterials with nucleic acids aiming for non‐viral gene delivery.^[^ ^46^, ^47^, ^48^, ^49^ ^]^ Thus, non‐viral, surface‐mediated gene delivery may represent an ideal strategy to control cell response in the close vicinity of an implant material avoiding any systemic complications in patients. Lipoplexes (LPX), a subtype of nucleic acid lipid nanoparticles, belong to the non‐viral gene delivery systems. For example, in a proof‐of‐concept study by Holmes, Lipofectamine 2000‐based LPX has been immobilized in PEMs and successfully transferred a model gene to typical screening cell lines (NIH3T3 fibroblasts and HEK293 kidney cells), but not to stem cells.^[^ ^50^ ^]^ However, we recently developed a LPX formulation composed of dioleylphosphatidylethanolamine (DOPE) and the ionizable lipid *N*‐{6‐amino‐1[*N*‐(9*Z*)‐octadec‐9‐enylamino]‐1‐oxohexan‐(2*S*)‐2‐yl}‐*N*′‐{2‐[*N*,*N*‐bis(2‐aminoethyl)amino]ethyl}‐2[(9*Z*)‐octadec‐9‐enyl]propan diamide (OO4), a lipid composite which demonstrated superiority in terms of efficient cellular uptake and DNA delivery in cell culture experiments, compared to Lipofecatmine 2000.^[^ ^51^, ^52^ ^]^ Moreover, we could show recently that surface coatings composed of either Col/Cs or Col/hyaluronic acid PEMs with dexamethasone (Dex) loaded OO4/DOPE liposomes could induce either osteogenic or chondrogenic differentiation of multipotent stem cells. This was related to the type of multilayer mimicking the composition of target ECM, such as bone or cartilage.^[^ ^53^, ^54^ ^]^ Furthermore, we developed in a proof‐of‐concept study, a strategy to incorporate LPX into PEMs composed of hyaluronic acid and chitosan and demonstrated the successful transfection of murine myoblasts and the epithelium of the chorion allantois membrane of the chicken embryo.^[^ ^48^ ^]^

**Figure 1:**
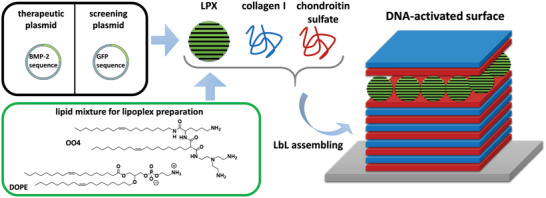
Schematic illustration of the DNA‐activated bone‐ECM‐mimicking surface coating. The lipid components OO4/DOPE were formulated to cationic liposomes. The cationic liposomes were assembled with DNA encoding either of the reporter gene green fluorescent protein (GFP) or the therapeutic gene BMP‐2, to LPX. LPX were assembled into DNA‐activated surface coatings as tool for in situ transfection using the LbL technique.

In the present study, we combined both approaches, such as the ECM‐mimicking character of PEM and their ability to be used as carrier for in situ transfection, to develop a gene‐activated ECM‐mimicking surface coating to direct stem cells’ fate. We focused on a bone ECM‐mimicking PEMs consisting of Col and Cs loaded with LPX composed of OO4/DOPE lipid composite (see **Figure** **1**). The DNA as biological active compound encoded a BMP‐2 sequence to stimulate surface‐mediated, transient expression of BMP‐2 in human adipose‐derived mesenchymal stem cells (hADSCs) to induce osteogenesis due to autocrine and paracrine effects of the cytokine. The work focused on three main objectives: 1) a material science part, in which we characterized the multilayer formation processes, especially the embedding of OO4/DOPE LPX and the surface properties of LPX‐loaded PEMs. 2) In addition, general studies on cell proliferation, hADSCs attachment, and transfection were performed. 3) We studied the ability of the system to induce osteogenic stem cell differentiation by gene expression analysis and mineralization assays. Summarizing, we present a new approach to engineer a bone‐ECM inspired gene‐activated surface coating which allows controlling stem cells function, and consequently, represents a promising tool to develop multifunctional surface coatings for regenerative medicine strategies.

## 2 Experimental Section

### 2.1 Materials

If not stated otherwise, all chemicals were purchased from Sigma–Aldrich/Merck (Taufkirchen, Germany). Col was provided from Sichuan Mingrang Bio‐Tech (Sichuan, China). The pDNA pCMV‐GFP (3.5 kbp, 260 kDa) was acquired from Plasmid Factory (Bielefeld, Germany). The synthesis and characterization of the used cationic lipid OO4 was described in the authors’ previous work.^[^ ^55^ ^]^ The phospholipids DOPE, 1,2‐dioleoyl‐*sn*‐glycero‐3‐phosphoethanolamin‐*N*‐(7‐nitro‐2‐1,3‐benzoxadiazol‐4‐yl) (ammonium salt) (NBD‐DOPE) (*λ* ^ex^_max_ = 460 nm and *λ* ^em^_max_ = 535 nm), and 1,2‐dioleoyl‐*sn*‐glycero‐3‐phosphoethanolamine‐*N*‐(lissamine rhodamine B sulfonyl) (ammonium salt) (Rho‐DOPE) (*λ* ^ex^_max_ = 560 nm and *λ* ^em^_max_ = 583 nm) were acquired from Avanti Polar Lipid, Inc. (Alabaster, AL, USA).

### 2.2 Methods

#### 2.2.1 Preparation of Cationic Liposomes

For liposome preparation, lipids were separately dissolved in chloroform/methanol (8:2, v/v) as lipid stock. The stocks were combined in the desired molar ratio (OO4/DOPE 1/3 *n*/*n*, OO4/DOPE/Rho‐DOPE 1/3/0.04 *n*/*n*/*n*, OO4/DOPE/NBD‐DOPE 1/3/0.04 *n/n/n*) and the organic solvent was evaporated for 1 h at 200 mbar at a rotary evaporator. After formation of dry lipid film, a solution of 150 mm NaCl 10 mm acetic acid adjusted to pH4 was added to a final total lipid concentration of 1 mg mL^−1^. Afterward, the lipid dispersion was incubated at 50 °C while shaking gently for 30 min at 1400 rpm (Eppendorf Thermomixer 5436) followed by sonication at 37 kHz and 50 °C for 5 min.

#### 2.2.2 Plasmid DNA Isolation

pDNA with the human BMP‐2 gene controlled by a human cytomegalovirus promoter controlled and containing a neomycin resistance gene (pCMV‐BMP‐2) was purchased from OriGene Technologies GmbH (Herford, Germany). It was cloned and amplified using *Escherichia coli* DH5*α* safety strain (Invitrogen, Carlsbad, CA, USA). Plasmid purification was performed using a Plasmid Maxi Prep Kit (Qiagen, Venlo, Netherlands) according to manufacturer instructions, and the resulting pDNA pDNA) was resuspended in MilliQ water. The pDNA concentration and purity were measured using a UV spectrophotometer at 260 and 280 nm and gel electrophoretic analysis.

#### 2.2.3 Lipoplex Formation

LPX were prepared by combining pDNA with OO4/DOPE 1/3 (*n*/*n*) liposomes to a N/P ratio (N = primary amines of the cationic lipids; P = phosphate groups of the nucleic acid) of 4 in sterile‐filtered solution of 150 mm NaCl with 10 mm sodium acetate buffer solution (pH 4). pDNA was pipetted to the liposomes and gently mixed, followed by an incubation period of 15 min at room temperature.

#### 2.2.4 Characterization of Lipoplexes and Liposomes

The size was determined by dynamic light scattering (DLS) and zeta potential by laser Doppler velocimetry (lDv) using a Zetasizer Nano ZS ZEN3600 (Malvern Panalytical, Malvern, UK) as described previously.^[^ ^53^ ^]^ Briefly, DLS measurements at a scattering angle of 173° consist of 15 runs with a duration time of 20 s for each. For size calculations, a viscosity *η* = 0.8872 mPa s and a refractive index of 1.33 were assumed. lDv was performed in a clear disposable folded capillary cell (DTS1060, Malvern Panalytical) with 30 runs at a voltage of 60 V. For data evaluation, the viscosity (*η* = 0.8872 mPa s), dielectric constant (*ε* = 78.5 F m^−1^), and refractive index (*n* = 1.33) of water were applied. Particle size distribution curves and zeta potential were calculated using Zetasizer Software 7.13 (Malvern Panalytical). All measurements were performed three times at 25 °C.

#### 2.2.5 Preparation of Polyelectrolyte Multilayers

The polyelectrolytes solutions were prepared as follows: Polyethylenimine (PEI, *M*_W_ ≈ 750 kDa) was dissolved in 0.15 m NaCl solution to a concentration of 5 mg mL^−1^ at pH 7.4. Cs (*M*_W_ ≈ 25 kDa) was dissolved in 0.15 m NaCl solution to a concentration of 0.5 mg mL^−1^ at pH 4. Col (*M_W_* ≈ 100 kDa) was dissolved in 0.2 m acetic acid to a concentration of 2 mg mL^−1^ (stirring overnight). The final solution of Col was obtained by diluting the stock solution in 0.2 m acetic acid supplied with 0.15 m NaCl at pH 4.

PEMs were assembled on surfaces (mainly glass coverslips, but also silicon wafers) cleaned using the RCA protocol.^[^ ^56^ ^]^ PEI was used as the first layer to obtain a positive charge on the substrate followed by adsorption of Cs as an anionic layer and afterward, Col as the cationic layer. PEMs were fabricated by immersing the glass coverslips or silicon wafers in the polyelectrolyte solution for 15 min (PEI, Cs) and 20 min (Col) and one layer of LPX for 2 h 30 min. Due to the different *M*_w_ of the polyelectrolytes, the larger Col molecules require more time for diffusion. For that reason, the adsorption time of Col was prolonged to 20 min following existing LbL protocols.^[^ ^57^ ^]^ By alternating adsorption of Cs and Col, a basal PEM consisting of four polyelectrolyte bilayers and a final Cs layer was fabricated [Cs/Col]_4_Cs, followed by LPX adsorption [Cs/Col]_4_Cs/LPX. Last, a Cs/Col cover layer was deposited to prepare the gene‐activated PEM [Cs/Col]_4_Cs/LPX/Cs/Col. Each adsorption step was followed by rinsing with 0.15 m NaCl solution at pH 4 (3 × 5 min).

#### 2.2.6 Confocal Laser Scanning Microscopy to Study Lipid and DNA Deposition on PEM

A rhodamine‐labeled lipid formulation of OO4/DOPE/Rho‐DOPE 1/3/0.04 (*n*/*n*/*n*) (*λ* ^ex^_max_ = 560 nm; *λ* ^em^_max_ = 583 nm) and Cy5 labeled pDNA (*λ* ^ex^_max_ = 649 nm; *λ* ^em^_max_ = 670 nm) was used to screen for LPX deposition on the PEMs.^[^ ^53^ ^]^ DNA was covalently tagged with Cy5 using the Label IT Nucleic Acid Labeling Kit from Mirus (Madison, WI, USA), according to the manufacturer's instructions. After production of [Cs/Col]_4_Cs/LPX using the fluorescence‐tagged LPX, the PEM was washed three times, and the Cs/Col cover layer was deposited to obtain the final construct [Cs/Col]_4_Cs/LPX/Cs/Col for microscopical analysis. The films were fixed with Aquatex mounting medium (Merck, Darmstadt, Germany) and stored overnight at 7 °C to cure the mounting medium before examining the distribution of the fluorophores by confocal laser scanning microscopy (CLSM) (LSM 710, Carl Zeiss, Oberkochen, Germany).

For time dependent evaluation of DNA embedding in the PEMs, the LPX were prepared with Cy5 labeled pDNA and embedded in the PEMs. The PEMs were stored in phosphate‐buffered saline (PBS) and Cy5 fluorescence was screened by CLSM at different time points using identical parameter settings for taking the micrographs. The fluorescence intensity was determined with 12 images per sample in triplicates using ImageJ.

#### 2.2.7 pDNA Loading Efficiency on the PEM Studied with Gel Electrophoresis

DNA loading of PEMs was quantified by agarose gel electrophoresis using an established protocol for indirect quantification (see for visualization of method also Figure S1, Supporting Information).^[^ ^48^ ^]^ After basal PEM [Cs/Col]_4_Cs preparation on coverslips in 24‐well plates, the coverslips were rinsed with 0.15 m NaCl (pH 4) and then transferred into a new 24‐well plate for the incubation with LPX. Various OO4/DOPE 1/3 (*n/n*) N/P 4 LPX concentrations (0.26 to 3.15 µg pDNA cm^−^^2^) were used for incubation under gentle shaking for 2 h 30 min. Subsequently, the supernatant of each well was transferred into tubes for quantification of DNA in LPX, and PEMs were afterward washed twice with 0.15 m NaCl solution (pH 4). The washing solutions were also transferred into separate tubes for quantification of DNA in detached LPX. DNA quantification was performed by gel electrophoresis after releasing the DNA from LPX. Briefly, 50 µL supernatant/washing solution was mixed with 10 µL of blue/orange 6× loading dye (G190A) and 4 µL 1% heparin v/w. Heparin was used to release complexed DNA from LPX. Electrophoresis was performed on 1% agarose gel containing 0.308 µg mL^−1^ Ethidium bromide in 1% Tris‐acetate‐EDTA buffer (pH 8) for 1 h at 90 V, while a 1 kb DNA ladder (G571A) (Promega, Madison, WI, USA) was used for size determination. Quantification was possible using a pDNA standard dilution series (0.01, 0.1, 0.2, 0.3, 0.5, and 0.8 µg pDNA) to obtain a calibration curve. The fluorescent DNA bands were quantified with a UVP UVsolo touch (Analytik Jena AG, Jena, Germany) for imaging and the software VisionWorks LS Analysis Software from Analytik Jena AG for fluorescence signal quantification. All samples were tested in triplicates. The sensitivity of the used method was <0.015 mg cm^−2^.

#### 2.2.8 Quartz Crystal Microbalance with Dissipation Monitoring

A Q‐Sense Pro quartz‐crystal microbalance with dissipation monitoring (QCM‐D, Biolin Scientific, Gothenburg, Sweden) was used to monitor LPX deposition on PEMs in detail. Freshly cleaned gold‐coated 5 MHz AT‐cut quartz crystal sensors (QSX301 Gold, Q‐Sense) were used as substrate for the build‐up of the PEM thin films. The solutions were injected into a flow chamber with a mounted quartz crystal at a constant flow rate of 50 mL min^−1^. The quartz crystal was excited at multiple overtones (1st, 3rd, 5th, 7th, 9th, 11th, and 13th, corresponding to 5, 15, 25, 35, 45, 55, and 65 MHz, respectively) and shifts in frequency (Δ*f_n_*) and energy dissipation (Δ*D*) were monitored in real‐time. The frequency of each overtone was normalized to the fundamental resonant frequency of the quartz crystal substrate (Δ*f_n_/n*, in which *n* denotes the overtone number). An adsorption time of 6 min for each polyelectrolyte layer and an intermediate rinsing step of 4 min with acetate buffer 0.1 m pH 5.5 were established. LPX solution was injected and measured for 2 h in steady state without a constant flow to mimic deposition conditions of the film preparation mentioned above. The hydrodynamic thickness of the PEMs at each deposition cycle as well as at the end of the deposition cycles was estimated using the Voigt‐based viscoelastic model implemented in the Q‐Sense Dfind software (Broadfit function), assuming a fluid density of 1000 kg m^−3^, a layer density of 1000 kg m^−3^, and a fluid viscosity of 1 mPa s.

#### 2.2.9 Water Contact Angle Measurements

Static water contact angle (WCA) measurements were analyzed at room temperature using an OCA15+ device from Dataphysics (Filderstadt, Germany). The sessile drop method was applied using 1 µL of water with the Ellipse‐fitting method. Reported contact angles represent mean values and standard deviation of five measurements per sample of duplicates.

#### 2.2.10 Atomic Force Microscopy

Atomic force microscopy (AFM, Nanowizard IV, JPK‐Instruments, Berlin, Germany) in combination with an inverted fluorescence microscope (OlympusIX71, Olympus, Olympus Europa, Hamburg, Germany) was performed in quantitative imaging mode (QI) to investigate the surface roughness and topography as well as record corresponding fluorescence images. Topographical images were recorded using a silicon cantilever (qp‐BioT, Nanosensors, Neuchatel, Switzerland) in a standard liquid cell (JPK‐Instruments) containing 0.15 m NaCl solution. A force map area of 5 × 5 µm2 was recorded with a resolution of 512 × 512 pixel2. Post‐processing and roughness analysis were performed using the software JKP Data Processing V5.0.85 and Gwyddion (GwyddionV2.58, 64‐bit).

#### 2.2.11 Fluorescence Recovery after Photobleaching

FRAP (fluorescence recovery after photobleaching) experiments using CLSM were performed to evaluate the LPX mobility in the PEM. This technique was developed by Axelrod et al. (1976) as a method to study mobility of substances for example proteins.^[^ ^58^ ^]^ In FRAP, a specific area is photobleached by intense laser light, removing fluorescence from this area, and screening afterward, the degree of fluorescence reappearance in this region. The used fluorophore for this study was NBD‐DOPE. The fluorophore was used to prepare fluorescence tagged liposomes (OO4/DOPE/NBD‐DOPE 1/3/0.04 *n*/*n*/*n*) which were applied for LPX formation with pCMV‐GFP. The fluorescence tagged LPX were adsorbed to [Cs/Col]_4_Cs basal PEMs for 2 h 30 min. The LPX‐loaded PEM was finalized with an additional cover layer of Cs/Col to [Cs/Col]_4_Cs/LPX/Cs/Col. FRAP studies of NBD‐LPX loaded PEMs were performed using an LSM 710 confocal microscope. A magnification of 40× with an oil objective was used for that experiment. A defined area in the PEM was photobleached (laser 488 nm, 20 cycles with a laser line attenuator transmission 100%). After defined periods of time, the area was examined for NBD‐ fluorescence using the same setup parameters. Images were processed with the ZEN2012 software (Carl Zeiss). The analysis of images to quantify RFU was performed with Image J.

#### 2.2.12 Cell Culture

Cryopreserved hADSCs (StemPro) were obtained from Thermo Fisher Scientific (Waltham, MA, USA) and thawed and grown in Dulbeccos's modified Eaflesss medium (DMEM) supplemented with 10% fetal bovine serum (FBS) and 1% antibiotic and antifungal solution at 37 °C (basal culture medium, BM) in a humidified 5% CO_2_/95% air atmosphere. Cells of almost confluent cultures were washed once with sterile PBS followed by treatment with 0.25% trypsin/0.02% EDTA at 37 °C for 3 min. Trypsin was neutralized with DMEM with 10% FBS, and the cells were re‐suspended in DMEM after centrifugation at 250 × *g* for 5 min. Last, the cells were seeded on PEMs‐coated glass coverslips with a cell density of 1 × 10^5^ cells per mL. Cells used in this study were from passage P1‐P6, and 50% of the culture media was changed three times a week.

For the purpose of osteogenic differentiation experiments, the pDNA pCMV‐BMP2, which encodes for BMP‐2, was used for LPX preparation. After the cells had reached 90% confluence, the medium was changed to induce the osteogenic differentiation. The cells were cultured in the osteogenic induction medium (OM) containing 0.1 µm Dex, 10 mm sodium *β*‐glycerophosphate (ß‐Gly), and 0.05 mm ascorbic acid‐2‐phosphate (ASC), in addition toBM as described above. For the positive control, the StemPro Osteogenic‐Differentiation Kit from Thermo Fisher Scientific (Waltham, MA, USA) was used according to the manufacturer's protocol. This medium contains components and cytokines for an optimized osteogenic differentiation of hADSCs and other stem cells provided by the supplier. As negative control group, the cells received BM. In addition, hADSCs cultured on [Cs/Col]_6_ in OM were used as LPX‐free positive control. The cells were incubated for 24 or 28 days and medium was changed once a week. All samples were tested for mineralization and gene expression of osteogenic markers (see below).

#### 2.2.13 Cell Adhesion Studies

Glass coverslips were coated with different PEMs composites: [Cs/Col]_6_ (LPX free system), [Cs/Col]_4_Cs/LPX (system with LPX on surface), and [Cs/Col]_4_Cs/LPX/Cs/Col/ (final LPX loaded PEM); placed in 24 well plates, hADSCs were seeded on the samples in DMEM at 37 °C for 4 h. Then, cells attached to PEMs were fixed with 4% paraformaldehyde solution for 10 min and rinsed twice with PBS for further studies. Cells were permeabilized with 0.1% Triton X‐100 in PBS v/v (Sigma) for 10 min, rinsed with PBS, and nonspecific binding sites were blocked by incubation with 1% w/v bovine serum albumin (BSA, Merck, Darmstadt, Germany) in PBS at room temperature for 1 h. Vinculin was stained using monoclonal anti‐vinculin clone hVIN‐1 mouse ascites fluid antibody (1:200, Sigma–Aldrich, Germany); and a secondary goat anti‐mouse IgG, Alexa Fluor 647 (1:1000, *λ* ^ex^_max_ = 650 nm and *λ* ^em^_max_ = 583 nm, Thermo Fisher Scientific, Waltham, MA, USA). The actin cytoskeleton was stained with phalloidin‐Atto 488 (1:50, *λ* ^ex^_max_ = 500 nm and *λ* ^em^_max_ = 520 nm, Sigma–Aldrich, Germany) at room temperature for 30 min. Cell nuclei were stained by BOBO‐1 Iodide (1:200, *λ* ^ex^_max_ = 462 nm and *λ* ^em^_max_ = 481 nm, Invitrogen, Darmstadt, Germany), incubating the samples for 30 min. Before microscopic evaluation, samples were washed with PBS and mounted with Roti‐Mount FluorCare (Carl Roth GmbH, Karlsruhe, Germany). Fluorescence micrographs were taken with a LSM 710 confocal microscope using 10×, 20× objectives for cell adhesion and spreading analysis. A 63× oil immersion objective was used to visualize nuclei, actin cytoskeleton, and focal adhesions. Images were processed with the ZEN2012 software (Carl Zeiss). The analysis of images to quantify cell count and cell area was performed with Image J.

#### 2.2.14 Cell Proliferation Studies with QBlue Cell Viability Assay

hADSC cells were seeded on LPX‐loaded PEMs with and without cover layer: [Cs/Col]_4_Cs/LPX; [Cs/Col]_4_Cs/LPX/Cs/Col. Cells seeded on clean glass coverslip and the LPX‐free PEMs [Cs/Col]_6_ were used as controls. Cultures were incubated at 37 °C for 24 h, 2 and 4 days, respectively. After the incubation time, the cell viability was determined by QBlue cell viability assay kit (Biochain, Hayward, NJ, USA). The cells were washed once with PBS to remove the medium. Then, 500 µL of Qblue solution with colorless medium (10:1) was added to each well and incubated at 37 °C for 3 h. Finally, 100 µL of supernatant from each sample was added to a black 96 well plate and the fluorescence intensity was measured at 544 nm excitation and 590 nm emission with plate reader (FLUOstar Omega, BMG Labtech, Ortenberg, Germany). All samples were tested in triplicates.

#### 2.2.15 DNA Uptake into Stem Cells

To visualize the DNA uptake from LPX‐loaded PEMs, we prepared fluorescence‐tagged LPX using Cy‐5 (*λ* ^ex^_max_ = 649 nm and *λ* ^em^_max_ = 666 nm) labeled pDNA‐GFP (Label IT Nucleic Acid Labeling Reagents, pDNA‐GFP labeled according to manufacturer's instructions) for the LPX preparation. After hADSCs were cultured for 48 h on the [Cs/Col]_4_Cs/LPX/Cs/Col coating, cells were screened for Cy‐5 positive structures while additional staining of nuclei and actin was performed for visualization of intracellular distribution of pDNA‐GFP. For this purpose, the cells were fixed, permeabilized, and blocked as described above. The order of cell staining was designed as follows: a) Phalloidin‐Atto 488 (1:50) for staining filamentous actin and b) BOBO‐1 (1:200) for staining the nucleus. All dyes were incubated for 30 min at room temperature and protected from light. PBS washing (three times, each 5 min) was performed after incubation with fluorescent dyes. Afterward, all samples were briefly washed with ultrapure water and mounted with Mowiol 4–88 containing 25 mg mL^−1^ 1,4‐diazabicyclo [2.2.2]‐octane (Carl Roth GmbH, Karlsruhe, Germany), a mounting medium providing high fluorescence stability for storage at 4 °C in the dark. Samples were analyzed with a LSM 710 confocal microscope.

#### 2.2.16 Flow Cytometry Measurements to Determine the Reporter Gene Expression

Cells with a cell density of 1 × 10^5^ cells per mL were seeded on PEMs containing pDNA‐GFP encapsulating LPX. After an incubation period of 24 h at 37 °C and 5% CO_2_, the expression of the reporter gene encoding for green fluorescent protein (GFP) was measured by flow cytometry. Briefly, cells were detached with 0.05% trypsin/0.02% EDTA solution from the PEMs and centrifuged at 220 × *g* for 5 min, rinsed, and re‐suspended in 500 µL of PBS containing 1% BSA. A BD Accuri C6 Plus flow cytometer (BD Bioscience, Franklin Lakes, NJ, USA) was used to analyze 10 000 cells per sample for GFP expression quantifying the relative fluorescence (GFP: *λ* ^ex^_max_ = 488 nm and *λ* ^em^_max_ = 510 nm). Single cells were gated by size (FSC‐H) and granularity (SSC). The calculated single cell population was gated to detect GFP‐expressing by calculating the relative number of transfected cells and dead cells. The BD Accuri C6 Software was used for all data evaluation. All samples were tested in duplicate.

#### 2.2.17 Mineralization Experiments

##### Alizarin‐Red‐Assay

After 24 days of the osteogenic differentiation experiments, calcium phosphate deposition was investigated by Alizarin Red S staining. Briefly, the samples were washed once with PBS and fixed with 4% paraformaldehyde for 10 min. After washing twice with distilled water, Alizarin Red S (2%, pH 4.2, Roth) solution was added into each well and incubated for 45 min under light exclusion at room temperature. Last, the excess dye was removed by washing with distilled water. Images were taken in transmission mode with a Nikon ECLIPSE Ti2, Tokyo, Japan equipped with a CCD camera (DCIN, Tokyo, Japan).

##### OsteoImage Kit

The commercial mineralization kit (OsteoImage, Lonza) was used to visualize the hydroxyapatite portion of bone‐like nodules deposited by cells by measuring fluorescence measurement (*λ* ^ex^_max_ = 495 nm and *λ* ^em^_max_ = 519 nm). This assay, as described by the manufacturer, uses a fluorescent staining reagent that binds specifically to the hydroxyapatite portion of the biomineralized structures. The intensity of the green fluorescence is proportional to the amount of hydroxyapatite in the sample. After 24 days of the osteogenic differentiation, samples were incubated with OsteoImage according to the manufacturer's instructions and examined with a LSM 710 confocal microscope.

#### 2.2.18 Gene Expression Analysis

After 28 days of differentiation, the mRNA was extracted from samples using Aurum Total RNA Mini Kit spin columns from BioRad (Hercules, CA, USA) according to the manufacturer's recommended procedure. First, strand cDNA was synthesized using an iScript Advanced cDNA Synthesis Kit for RT‐qPCR (real‐time quantitative polymerase chain reaction, Biorad) in 20 µL reactions, according to the manufacturer's instructions. A CFX Connect RT‐qPCR Detection System (Biorad and pre validated primer sets PrimePCR Probe Assays from Biorad were used for gene expression analysis of the transcription factor Noggin (assay ID: qHsaCEP0054879), Collagen type 1 alpha 1 (Col1A1; assay ID: qHsaCEP0050510), Run‐related transcription factor 2 (RunX2; assay ID: qHsaCEP0051329), Alkaline Phosphatase (ALP; assay ID: qHsaCEP0024224), and BMP‐2 (assay ID: qHsaCIP0029912). RPLP0 (assay ID: qHsaCEP0041375) was used as housekeeping gene. Data analysis was performed using the BioRad CFX Manager Software 3.0. The following scheme was used for the RT‐qPCR: 95 °C for 30 s followed by 39 cycles at 95 °C for 15 s and 60 °C for 30 s. The relative expression levels of each gene were calculated and normalized to the housekeeping gene RPLP0 using the DDCt method (2^−ΔΔ*Ct*^.).^[^ ^59^ ^]^

#### 2.2.19 Statistical Analysis

All statistical analyses were performed with Origin 8G software. If not stated otherwise, experiments were performed in triplicates (*n* = 3) and the results presented as means +− standard deviation (SD). If the number of experiments *n* was different from 3, the value of *n* was given in the caption. Analysis of significance was performed by one‐way ANOVA followed by Scheffé post hoc test with *α* = 0.05. A value of *p* < 0.05 was considered as a significant difference and was indicated by an asterisk. Further, box plots are shown where appropriate. The box indicates the 25th and 75th percentiles, the median (dash), and mean value (black square), respectively.

## 3 Results and Discussion

### 3.1 Characterization of Lipoplexes

**Figure 2:**
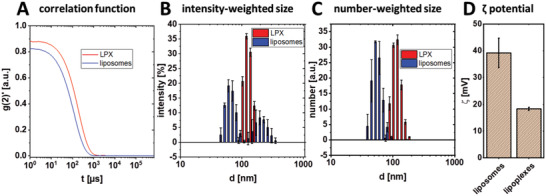
Correlation functions (representative of three measurements) A) of DLS measurements and the resulting B) intensity‐weighted and C) number‐weighted size distribution curves of OO4/DOPE liposomes (blue line) and LPX (0.1 µg pDNA, N/P 4) (red line) in 0.15 m NaCl containing 10 mm sodium acetate buffer at pH 4. D) Zeta potential results of OO4/DOPE liposomes and LPX. Results are means and standard deviations of three measurements (B–D).

The lipid formulation OO4/DOPE 1/3 (*n*/*n*) (hereinafter referred to as OO4/DOPE) was used to prepare LPX as transfection active component for the gene‐activated surface coating. OO4 is a cationic peptide‐mimicking amphiphile designed in our group^[^ ^43^, ^55^ ^]^ and provided the positive charge for DNA complexation as well as PEMs assembly. DOPE is a commonly used co‐lipid for lipid‐based transfection agents. For efficient immobilization of LPX into PEMs via electrostatic interaction, a positive net charge was essential. DLS and zeta potential measurements were carried out to obtain information on particle size and charge of OO4/DOPE liposomes and, more substantially, of LPX under LbL preparation conditions. The data are presented in **Figure** **2**. The autocorrelation function (Figure 2A) was characterized by intercepts at 0.9 for LPX (red) and 0.8 for liposomes (blue), a sigmoidal decay of the signal and the absence of a noisy baseline, indicating a good quality of DLS data for reasonable fitting. For the liposomes, the intensity‐weighted size distribution showed a bimodal function, with a particle size population at diameter (*d*) ≈ 50–100 nm and *d* ≈ 200–400 nm (Figure 2B). For multimodal size distributions, the intensity‐weighted curve can be misleading because the scattering intensity is proportional to ≈*d* ^6^_._ Hence, small numbers of larger particles can dominate the distribution function. Therefore, the number‐weighted size distribution curves were calculated (Figure 2C), showing that the 50–100 nm population was in a much higher quantity than expected from the intensity‐weighted curve (compare blue size distribution curves). In contrast, LPX showed an unimodal size distribution with d ≈ 100–200 nm, in both intensity‐ and number‐weighted curves (Figure 2B,C). The observed changes in particle size of LPX, compared to the liposomes, are probably due to the lipid membrane reorganization process during the complex formation between DNA and the cationic liposomes, based on the templating effect of DNA.^[^ ^60^, ^61^ ^]^ Comparing zeta potential measurements of liposomes and LPX, a decrease in the zeta potential from *ζ* ≈ 38 mV to *ζ* ≈ 18 mV was observed (Figure 2D) due to complex formation between the positively charged liposomes and the negatively charged DNA. Nevertheless, the positive net charge of the LPX for embedding into PEMs was proven.

### 3.2 Structural Characterization of PEMs with Focus on LPX Embedding

**Figure 3:**
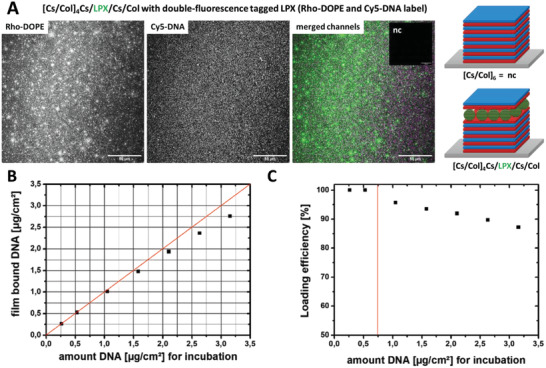
A) CLSM micrograph (40× magnification) of [Cs/Col]_4_Cs/LPX/Cs/Col PEMs with fluorescent tagged LPX. The following fluorescence labels were used: Rho‐DOPE as lipid label (left image in grey scale, green in the merged right image) and Cy5‐labeled pDNA (middle image grey scale, magenta in the merged image right), which were used to visualize LPX attached to the PEMs. The inlay on the merged image (right), labeled with nc = negative control, was the LPX‐free negative control [Cs/Col]_6,_ which was examined under the same conditions as proof for the absence of auto‐fluorescence effects of the polyelectrolytes. The scale bars indicate 50 µm. Images were analyzed using ImageJ. B) Schematic illustration of the PEM sequence codes [Cs/Col]_4_Cs/LPX/Cs/Col (sample) and [Cs/Col]_6_ (negative control). C) Film‐bound amount of DNA in µg cm^−2^ in [Cs/Col]_4_Cs/LPX/Cs/Col films as a function of the total DNA concentrations in the incubation medium (0.26–3.15 µg cm^−2^ LbL substrate). The calculation of the film‐bound DNA was based on the indirect quantification of the non‐bound DNA using agarose gel experiments presented in Figure S2, Supporting Information (*n* = 3). The red line shows the theoretical values of 100% binding efficiency. D) The calculated DNA loading efficiency from (C). Above is an LPX incubation with total DNA amount of 0.75 µg cm^−2^ PEM substrate (indicated by the red line); the loading efficiency decreases below 100%. (B,D) show the means ± SD of triplicates. The bars indicate that the SD are below 0.04 µg cm^−2^; and therefore, are not seen due to resolution of the graphs.

The embedding of LPX into PEMs composed of Cs and Col was demonstrated by CLSM using dual fluorescence‐tagged LPX: The pDNA was covalently labeled with Cy5 and the lipid composite was modified with 1.25‐mol% Rho‐DOPE as fluorescence label (**Figure** **3**A). The images indicate a homogeneous distribution of the labeled DNA as well as the lipid in the PEM [Cs/Col]_4_Cs/LPX/Cs/Col, considering the film curvature which leads to out‐of‐focus effects in the edge regions. Nevertheless, both channels do not fit in all details (merged images Figure 3A), an observation which can be explained by the fact that at a lipid‐DNA loading ratio of N/P 4, some DNA‐free cationic liposomes exist besides LPX.^[^ ^62^ ^]^ The LPX‐free control [Cs/Col]_6_ (Figure 3A, insert labeled with nc = negative control) shows no autofluorescence of the polyelectrolytes using the same experimental setup.

The DNA loading efficiency of [Cs/Col]_4_Cs/LPX/Cs/Col films was determined in more detail. It was not possible to quantify DNA embedded in PEMs directly by gel electrophoresis or fluorescence passed assays in a reproducible manner. The appearance of colloids after disintegration of LPX‐loaded PEMs, resulting in quenching and light‐scattering effects,^[^ ^63^ ^]^ may explain this problem. Recently, we described a method for an indirect quantification of DNA‐LPX loading into PEMs by quantifying the fraction of DNA which was not incorporated in PEMs.^[^ ^48^ ^]^ For this purpose, the DNA of the supernatant and washing solutions, which was most likely complexed in LPX, was quantified via gel electrophoresis, a method which needed a pre‐incubation with heparin to release all DNA from LPX (for details, see Figures S1 and S2, Supporting Information). The results are presented in Figure 3B,C. The incorporation of different amounts of DNA, from 0.26 to 3.15 µg cm^−2^, was evaluated. Up to a loading amount of ≈0.75 µg cm^−2^, the LPX can be efficiently incorporated into the LbL system (loading efficiency of 100%, Figure 3C). PEMs with concentrations above 0.75 µg cm^−2^ resulted in loading efficiencies below 100% (Figure 3C). In other studies on polyplex‐loaded PEMs, a much lower DNA content of 25–30 ng cm^−2^ was described, showing that PEMs consisting of Cs and Col and loaded with LPX represent an excellent system for gene‐activated PEMs.^[^ ^64^ ^]^ To evaluate whether LPX are desorbed from the PEM during subsequent rinsing steps, the washing solutions were also examined for DNA content, showing no burst release of adsorbed LPX or released DNA from the PEMs (for details, see Figure S2, Supporting Information).

**Figure 4:**
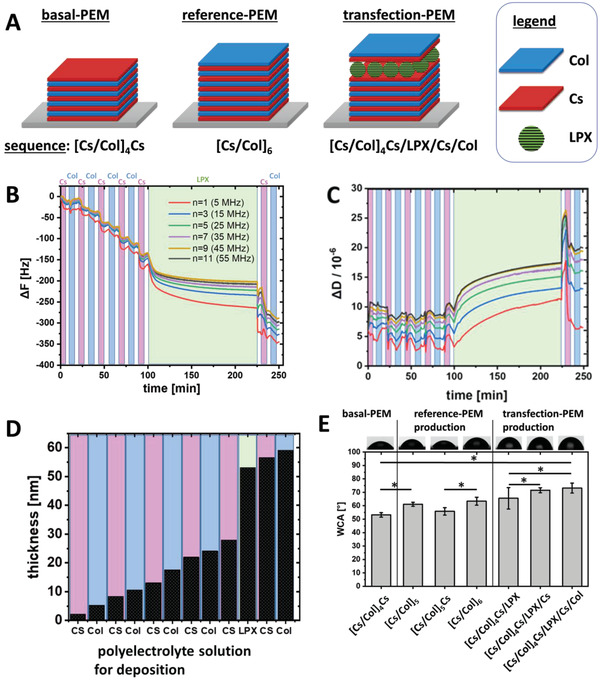
A) Schematic illustration of the LbL sequence codes of investigated PEMs. B–D) Build‐up of [Cs/Col]_4_Cs/LPX/Cs/Col multilayered thin films onto Au‐plated quartz crystal sensors via incubation with polyelectrolyte solutions to achieve LbL deposition. QCM‐D monitoring of the normalized frequency (ΔF, panel B) and dissipation (ΔD, panel C) shifts. The frequency shifts of different overtones are presented. The dissipation shift is shown for the 7th overtone (35 MHz). The background color indicates the incubation/washing medium: magenta = Cs; blue = Col; green = LPX; white = washing buffer (B,C). Cumulative hydrodynamic thickness evolution for the [Cs/Col]_4_Cs/LPX/Cs/Col PEM production, obtained using the Voigt‐based viscoelastic model (D). E) Static WCA measurement of intermediate and final PEM structures. The *x*‐axis demonstrates the film composition deposited as sequence code. Results represent means ± SD with *n* = 10; significance was tested using one‐way ANOVA followed by Scheffé post hoc test, *α* = 0.05, *p ≤ 0.05.

QCM‐D measurements were performed to monitor the material deposition during the LbL assembly process in situ. In **Figure** **4**B, the frequency shift of different overtones is plotted. The stepwise decrease of ∆*F* indicates the successful deposition of material, Cs, Col, or LPX, respectively, after each incubation step. Multilayer growth was thus proven for each deposition step. The interaction of the charged biopolymer or LPX with the oppositely charged surface was effective under the chosen assembly conditions with a pH value of 4 and 150 mm NaCl as electrolyte solution. The time to reach the adsorption equilibrium was much longer for LPX when compared to Cs and Col. Nevertheless, the time periods needed for deposition provide evidence that we reached the adsorption equilibrium with the PEM preparation protocol used in this study (Section 2.2.5). The following rinsing step did not result in an increase of ∆*F*; thus, excluding the eventual desorption of adsorbed LPX. The evaluation of the dissipation changes demonstrated a pronounced increase in ∆*D* during LPX adsorption (Figure 4C, green area). Obviously, the plastic proportion in the viscoelastic behavior increased, concluding that the PEM gets softer and more hydrated due to the adsorption of LPX. The high ∆*D* value remained also after the deposition of the Cs and Col cover layers on the PEM film (final sequence [Cs/Col]_4_Cs/LPX/Cs/Col). Obviously, the LPX dominated the film mechanics even with the outermost Cs/Col layers. Previous structural investigation has demonstrated that the LPX composed of OO4/DOPE are soft matter nanoparticles with a liquid crystalline substructure;^[^ ^65^ ^]^ consequently, a viscoelastic behaviour was expected. Furthermore, the cumulative hydrodynamic thickness evolution during the construction of the multilayer film was calculated from QCM‐D data (Figure 4D). A linear increase of thickness was observed for the deposition of either Cs or Col. The thickness increase of ≈30 nm after LPX deposition was much higher compared to the biopolymers. Nevertheless, the DLS size distribution curves (Figure 2B,C) resulted in a main LPX diameter of 100–150 nm, a much higher value. Two facts may explain the discrepancy: 1) The LPX do not cover the whole area (we have evidence for that conclusion from CLSM micrographs; see also Figure 3A) combined with the fact that the QCM‐D determined thickness is a mean thickness. 2) As mentioned above, LPX are soft matter nanoparticles, and a deformation and flattening of the LPX nanoparticles after adsorption on the surface can be expected.

The wetting behaviour of surfaces is an important parameter because moderate wettable surfaces with WCA ≈60° promote protein adsorption and cell adhesion.^[^ ^66^ ^]^ Figure 4E shows the WCA of selected intermediates and the final construct of the DNA‐activated PEM and the LPX‐free reference PEM of comparable layer number. Starting from the basal‐PEM [Cs/Col]_4_Cs, which was the substrate for the LPX adsorption, a WCA of ≈53° was measured, indicating a moderate wettability related to the presence of Cs as more hydrophilic molecule with carboxylic and sulfate groups.^[^ ^67^ ^]^ When LbL was continued with Col adsorption (sequences [Cs/Col]_5_), a WCA of ≈61° was detected, which is related to additional presence of amino groups in the protein which are less wettable than anionic groups. The alternating WCA lower for anionic Cs and higher for Col terminal layers indicates the dominance of these molecules in the outermost layer of PEMs.

The adsorption of LPX (PEMs sequence [Cs/Col]_4_Cs/LPX) resulted in an increase of WCA to ≈66° compared to the previous WCA of ≈53° terminal Cs layer which is related to the cationic nature of the LPX. A further coating with Cs (PEM sequence [Cs/Col]_4_Cs/LPX/Cs) caused an increase of WCA to 72° which may be related to some structural rearrangements of LPX upon contact with Cs which may be related to the presence of hydrophobic parts of lipid species, observed also in a previous study with liposomes.^[^ ^53^ ^]^ The terminal Col coating [Cs/Col]_4_Cs/LPX/Cs/Col resulted in no significant change in WCA (≈73°) which indicates some intermingling of LPX layer with Cs and Col in the terminal layer related also to the much larger size of LPX compared to both polyelectrolytes.^[^ ^54^ ^]^

**Figure 5:**
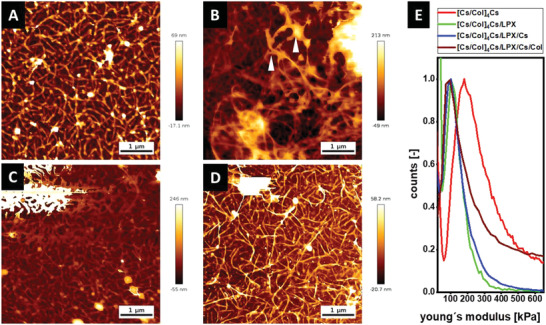
A–D) Topography images [Cs/Col]_4_Cs (A), [Cs/Col]_4_Cs/LPX (B), [Cs/Col]_4_Cs/LPX/Cs (C), and [Cs/Col]_4_Cs/LPX/Cs/Col determined by AFM [Scale bar 1 µm] (D). E) Distribution curves of Young's modulus (*E*_0_) with a force map of a defined area (see also Figure S4, Supporting Information).

AFM was performed to study the surface topography and mechanical properties of the different PEMs (**Figure** **5**). To investigate the elasticity and the force curve, the samples were compressed by the AFM tip and the force mapping mode was applied while the tip scanned a specific area of the sample.^[^ ^68^ ^]^ The force mapping mode measured the interaction forces such as adhesion or electrostatics and gives an idea regarding the stiffness and topography. The interest in testing mechanical properties and topography of surface coatings is related to their effect on cell behavior, such as spreading, proliferation, and differentiation.^[^ ^69^, ^70^ ^]^ In addition, intermediate LbL process steps were investigated to learn more about the LPX deposition and the embedding process. The basal‐PEM [Cs/Col]_4_Cs, which was the substrate for LPX adsorption, showed a homogeneous nanofibrous network that can be assigned to Col deposition and fibrillization with a roughness of ≈12 nm (Figure 5A, **Table** **1**). Previous studies demonstrated that Col/Cs PEMs are characterized by a fibrillary structure of Col.^[^ ^57^, ^67^, ^71^ ^]^ Due to its high charge density, Cs is known to promote the Col self‐assembly to fibrils.^[^ ^72^ ^]^ After the adsorption of LPX, the surface topography changed showing a less organized fibrillary substructure (Figure 5B) but an increase of the roughness ≈23 nm (Table 1). Diffuse structures of different sizes were observed for the [Cs/Col]_4_Cs/LPX film. In addition, the remaining fibrous structures appeared with a larger thickness of higher variability and more extended smeared structures (Figure 5B, arrowhead). The addition of a covering Cs layer induced the reappearance of the fibrillary topography with a roughness of ≈15 nm (Figure 5C, Table 1). The final DNA activated PEMs [Cs/Col]_4_Cs/LPX/Cs/Col was characterized by an extended fibrillar structure, comparable to the basal‐PEM (compare Figure 5A with Figure 5D; Figure S3, Supporting Information). The addition of the soft matter nanoparticles such as LPX might modify the arrangement of the Col fibers and it can influence the roughness. The behavior is also expressed by the roughness parameters shown in Table 1. *R*_a_ and *R*_q_ increased after LPX adsorption ([Cs/Col]_4_Cs → [Cs/Col]_4_Cs/LPX) but decreased again to the initial values for the full PEMs. The same tendency can be seen in the waviness parameters R3z ISO, *W*_a_, and *W*_q_ (Table S1, Supporting Information).

**Table 1:** Roughness parameters of area mean roughness (*R*_a_), area root mean squared roughness (*R*_q_) of PEMs sequences before and after LPX deposition. 1D roughness analysis, according to DIN EN ISO 4287, 4288, 3274, mean values ± SD calculated from ten separate lines, ln = 5 µm, Dc = 1 µm, cutoff filter: 0.02 measured by AFM

	*R*_a_ [nm]	*R*_q_ [nm]	*E* modulus [kPa]
[Cs/Col]_4_Cs	9.6 ±0.9	12.2 ±1.3	199.5 ± 6.3
[Cs/Col]_4_Cs/LPX	18.6 ±2.7	23.5 ±3.3	111 ± 3.1
[Cs/Col]_4_Cs/LPX/Cs	12.0 ±1.8	15.5 ±2.4	103 ± 3.5
[Cs/Col]_4_Cs/LPX/Cs/Col	9.4 ±1	12.8 ±1.8	98 ± 4.5

Figure 5E presents the *E*_0_ modulus distribution curves of the highest and lower points in the measured area. The sequence [Cs/Col]_4_Cs was characterized by a broad distribution with a maximum value at *E*_0_ = 199 kPa while a minimum value was seen above 50 kPa. The mechanical properties of the basal layer show the Col fibers with higher stiffness (maximum) than the surrounding area (minimum). It is known that highly negatively charged polysaccharides such as Cs have interfusing characteristics in PEMs and can act as a cross‐linker for Col, supporting also the organization of fibrils and making these areas of PEMs stiffer.^[^ ^53^ ^]^ The adsorption of LPX reduced the stiffness of the PEMs showing a shift of *E*_0_ distribution curve to a maximum value of 111 kPa for [Cs/Col]_4_Cs/LPX. An explanation is that LPX represent liquid crystalline soft matter particles, which can reduce the stiffness.^[^ ^65^ ^]^ It is interesting to note that further adsorption of Cs, and then, Col caused a further decrease of *E*_0_ to 103 and 98 kPa, respectively. The lower *E*_0_ values of PEM after LPX adsorption and the additional bilayer of Cs/Col are also related to an increased thickness of PEMs that will further reduce the E modulus of the coating due to swollen character of PEMs fabricated from biopolymers.^[^ ^73^ ^]^ Furthermore, this observation is also in line with the effect of the LPX observed for the ∆*D* values discussed in the QCM‐D section above.

In summary of WCA and AFM studies, the complete PEMs [Cs/Col]_4_Cs/LPX/Cs/Col is characterized by moderate wettability and a higher roughness promoting cell adhesion.^[^ ^74^ ^]^ What's more, a decrease in the stiffness of PEM was observed when LPX and the additional Cs/Col bilayer were added; the observed E modulus in the range of 100 kPa was still supporting cell attachment and spreading as found in other studies.^[^ ^69^ ^]^

### 3.3 Mobility of LPX Incorporated in PEMs

**Figure 6:**
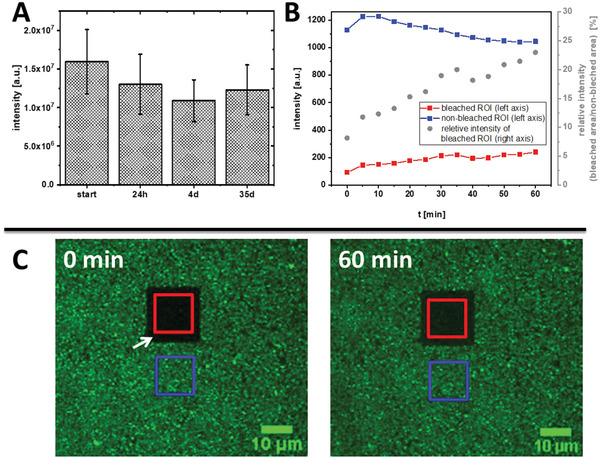
A) Fluorescence intensity of covalently labeled Cy5 DNA in LPX immobilized in PEM [Cs/Col]_4_Cs/LPX/Cs/Col. CLSM‐based intensity determination was done directly after the film construction (start) and after 1, 4, and 35 days. The results are given as means ± SD (*n* = 36). Significance was tested using one‐way ANOVA followed by Scheffé post hoc test, *α* = 0.05, *p ≤ 0.05, no significant difference was found. B,C) Results of FRAP studies. Fluorescence intensity of NBD‐DOPE was used as fluorescence probe in LPX (1 mol‐% of the lipid composite). A defined area was bleached with high laser intensity and micrographs for intensity determination were taken in a period of 60 min after bleaching. Images were analyzed using ImageJ (B). CLSM images of [Cs/Col]_4_Cs/LPX/Cs/Col loaded with NBD‐labeled LPX directly (0 min) and 60 min after bleaching. The white arrow indicates the bleached region which appeared black due to successful fluorophore inactivation. The red square indicates the area applied to the fluorescence intensity determination of the bleached region in diagram (B); the blue square indicates the reference area of a non‐bleached region (C).

Potential time dependent changes of the LPX layer in the PEMs [Cs/Col]_4_Cs/LPX/Cs/Col stored in PBS were studied using CLSM. Although because of their relatively large size and the electrostatic interaction with the polyanion Cs, a fast diffusion of LPX in the multilayer system was not expected. Nevertheless, two different experiments were performed to investigate if LPX have a certain mobility in PEMs. In the first experimental setup, Cy5 labelled DNA was used for the preparation of LPX which were afterward embedded in the PEMs. These samples were examined regarding the Cy5 fluorescence intensity at different time points of incubation in PBS by CLSM: directly after PEMs construction, as well as 24 h, 4 days, and 35 days after fabrication of PEMs. The results are shown in **Figure** **6**A. A weak trend of a decrease in Cy5 fluorescence was observed, but all detected differences were not statistically significant. Therefore, it can be assumed that the DNA of the LPX remained entrapped in the PEMs within the time interval of 35 days. The large size of DNA (3.5 kbp, 260 kDa) in LPX and the complexation with the cationic lipids are certainly the reasons for the stability of the system.

In the second experimental setup, FRAP experiments were performed (Figure 6B,C). Here, the lipid components of LPX were fluorescence tagged. NBD‐DOPE as efficiently bleachable fluorophore was used for the LPX preparation. Then, the PEM with embedded LPX was bleached at an area by applying high laser intensity (red box, Figure 6C). This photobleached area was examined for recovery of NBD fluorescence for 60 min. Indeed, a weak but steady increase in NBD fluorescence was detected in the bleached area. However, only ≈10% of the intensity of the non‐bleached reference area reappeared after 60 min (Figure 6B, red symbols). Note that the fluorescence in the control region was slightly decreasing (Figure 6B, blue symbols), an effect most‐likely assigned to photo bleaching. This would imply that the calculated relative intensity change (Figure 6B, blue symbols) is biased, and the real reappearance is lower. Hence, only a small fraction of non‐bleached fluorophores diffused into the bleached region, indicating that lipid components of LPX have certain mobility. Smaller molecules can diffuse in PEMs, as for example demonstrated for small model peptides.^[^ ^75^ ^]^

Summarizing, it can be concluded that the DNA is stably entrapped in LPX which will also be evident by the subsequent transfection studies.

### 3.4 Cell Studies

#### 3.4.1 Cell Adhesion and Proliferation of Mesenchymal Stem Cells on LPX‐Loaded PEMs

**Figure 7:**
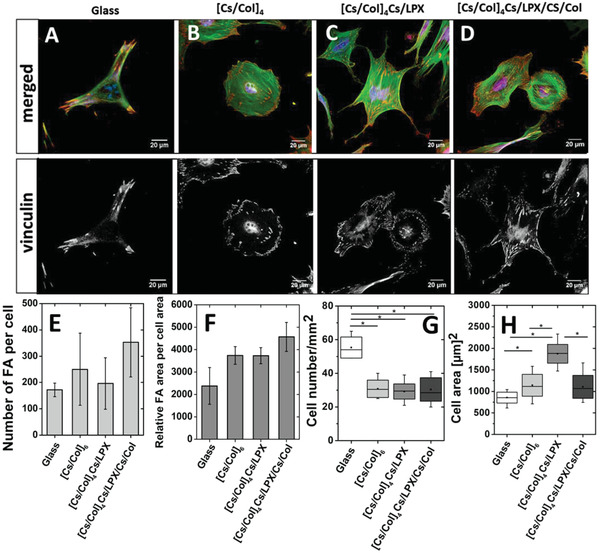
A–D) Merged CLSM image of adherent hADSCs cultured on the different PEMs after 4 h of incubation in serum. Glass slide (A), [Cs/Col]_6_ (B), [Cs/Col]_4_Cs/LPX (C), and [Cs/Col]_4_Cs/LPX/Cs/Col (D). The cells were stained for filamentous actin (green), vinculin‐positive FA (red), and nuclei (blue) in the merged images. The vinculin channel is shown separately in gray scale below the merged image. The scale bar represents 20 µm. E) Gives the quantification of vinculin‐positive FA number per cell and F) relative vinculin‐positive FA area per cell determined by Image J for representative six cells (*n* = 6; significance was tested using one‐way ANOVA followed by Scheffé post hoc test, *α* = 0.05, *p ≤ 0.05, no significant difference was found). G) Quantification of cell count per square millimeter and H) cell spreading area (µm^2^) on each of the PEMs after 4 h (*n* = ten images per condition). (Box plots with whiskers, representing first and third quartiles, medians and means. The star (*) indicates statistically significant differences using one‐way ANOVA followed by Scheffé post hoc test, *α* = 0.05, with a *p*‐value ≤ 0.05 (F,G).

Interaction of hADSCs with PEMs substrates was studied by staining nuclei used for cell counting, actin cytoskeleton used for cell spreading analysis, and the evaluation of focal adhesion (FA) as marker of cell‐ECM contact points. Cells cultured on glass as positive control showed an extended phenotype with longitudinal organization of actin filaments and well‐developed vinculin‐positive FA in the cell periphery, indicating a normal behavior of these cells (**Figure** **7**A, actin fibers shown in green). Cells cultured on LPX‐free PEMs [Cs/Col]_6_ were characterized by a longitudinal distribution of the actin filaments as well (Figure 7B, actin fibers shown in green). Many vinculin‐positive FA were detected (Figure 7B, red signal in the merged image and signal in the lower single channel image). Cells on the intermediate PEM construct [Cs/Col]_4_Cs/LPX were less extended, but also, a longitudinal organization of actin filaments was observed (Figure 7C, actin fibers shown in green). Slightly fewer and larger vinculin‐positive FA were predominantly observed at the cell periphery (Figure 7C, red signal in the merged image and signal in the lower single channel image). Cells cultured on PEMs [Cs/Col]_4_Cs/LPX/Cs/Col showed spread hADSCs with parallel arrangement of actin filaments (Figure 7D, green staining) and extended cell protrusions. Distinct vinculin‐positive FA were observed at the cell protrusions and cell periphery (Figure 7D, red staining merged image and signal in the lower single channel image). The quantitative evaluation of FA showed no statistically significant differences between the positive control and PEMs; for both, the number of FA per cell and the relative area of vinculin positive FA. In addition, no statistical difference was found between the different PEMs. However, a trend to higher values was observed for PEMs [Cs/Col]_4_Cs/LPX/Cs/Col (Figure 7E,F). The higher number of cells on the positive control in comparison to all PEMs shown in Figure 7G is probably related to the stiffness of glass that promotes cell attachment.^[^ ^76^ ^]^ On the other hand, the quantification of the cell area (Figure 7H) demonstrated that hADSCs on glass had a smaller cell area in contrast to [Cs/Col]4Cs/LPX PEM that showed higher cell spreading (Figure 7H). The determined cell areas for the reference PEMs [Cs/Col]_6_ and [Cs/Col]_4_Cs/LPX/Cs/Col were comparable and in between the values of glass and [Cs/Col]4Cs/LPX PEM. As is known, cell spreading is a promoter of osteogenic differentiation so the PEMs made of Cs/Col may support osteogenesis of hADSCs.^[^ ^77^ ^]^ Moreover, the presence of Col as component of all PEMs can be considered as a promoter of mitogenic signal transduction through integrin receptors such as *α*2*β*1 integrin, the main receptor for Col.^[^ ^59^ ^]^

**Figure 8:**
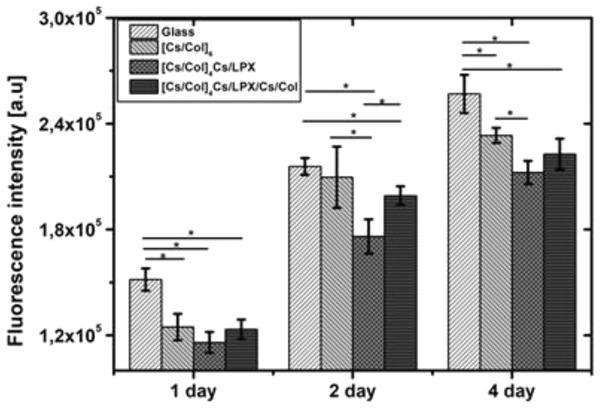
Growth of hADSCs seeded on PEMs sequence of glass slide (control), [Cs/Col]_6_, [Cs/Col]_4_Cs/LPX, and [Cs/Col]_4_Cs/LPX/Cs/Col measured by the QBlue assay after 24, 48, and 96 h. The values represent means ± SD with *n* = 3. The star (*) indicates statistically significant differences using one‐way ANOVA followed by Scheffé post hoc test, *α* = 0.05, with a *p*‐value ≤ 0.05.

The proliferation of hADSCs was studied by QBlue assay evaluating metabolic activity of cells seeded on glass slides (positive control), PEMs [Cs/Col]_6_, [Cs/Col]_4_Cs/LPX, and [Cs/Col]_4_Cs/LPX/Cs/Col. In **Figure** **8**, it is shown that cells seeded on glass showed higher metabolic activity in contrast to PEMs, which relates to the results of adhesion studies. For all three surface coatings, a significant increase of the metabolic cell activity from day 1 to day 4 was observed (Figure 8) indicating cell growth with no differences among them on day 1. Comparing the cell growth on subsequent days, it was seen that [Cs/Col]_6_ provided superior conditions while the presence of LPX in the two other systems had a slightly inhibiting effect on cell growth with significantly lower values comparing [Cs/Col]_4_Cs/LPX with [Cs/Col]_6_. This may be related to the cationic nature of LPX that may exert certain toxicity though adhesion studies did not provide any hints for that.^[^ ^51^ ^]^ Overall, all PEMs enabled attachment, spreading and growth of cells, as a prerequisite for subsequent cell differentiation studies.

#### 3.4.2 Transfection Studies

**Figure 9:**
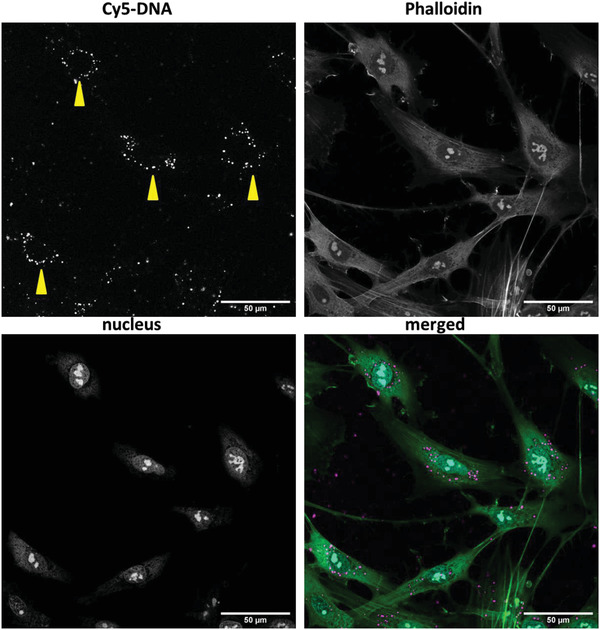
CLSM micrograph of transfected hADSCs after 48 h grown on [Cs/Col]_4_Cs/LPX/Cs/Col with Cy‐5 labeled DNA (merged image magenta). The cells are stained for filamentous actin with Phalloidin‐Atto 488 (merged image green) and nucleus with BOBO‐1 (merged image cyan). Images were taken at 40× magnification and evaluated with ImageJ. The bar represents 50 µm. Images are given as single channels and merged. The provided image is an optical cross section taken by CLSM analysis. An additional optical cross section at lower *z*‐value near the PEMs surface is given in Figure S5, Supporting Information.

Successful transfer of DNA from [Cs/Col]_4_Cs/LPX/Cs/Col into hADSCs is essential for a clinical in situ transfection strategy. To study the cellular uptake, LPX were loaded with Cy5 labeled DNA. The fluorescent tagged LPX were embedded in the [Cs/Col]_4_Cs/LPX/Cs/Col multilayer. Afterward, hADSCs were seeded onto the fluorescent tagged in situ transfection system and cells were evaluated after 2 days by CLSM (**Figure** **9**). Cellular uptake of the Cy5 labeled DNA was demonstrated, showing a Cy5 fluorescence signal accumulated in the perinuclear region (Figure 9, yellow arrows). However, it is described in the literature that the Cy5 label preferentially tends to accumulate in mitochondria because of their higher mitochondrial membrane potential compared to normal cells, which is why DNA is hardly recognizable in the cell nucleus.^[^ ^78^, ^79^ ^]^ The mechanism of LPX‐uptake by cells from the PEMs [Cs/Col]_4_Cs/LPX/Cs/Col is not understood in detail. However, a cell‐mediated endocytosis can be assumed despite the presence of a cover layer because it was demonstrated previously that mesenchymal stem cells can actively remodel Col of Cs/Col‐based PEMs.^[^ ^57^ ^]^ Moreover, we recently described successful endocytosis of liposomes from CS/Col‐based PEMs into C2C12 myoblasts adhering to the coating.^[^ ^54^ ^]^ It is further known that endocytotic uptake is the main route for LPX into cells.^[^ ^62^, ^80^ ^]^ To proof efficient DNA transfer to the nucleus, reporter gene transfection experiments were also performed.

**Figure 10:**
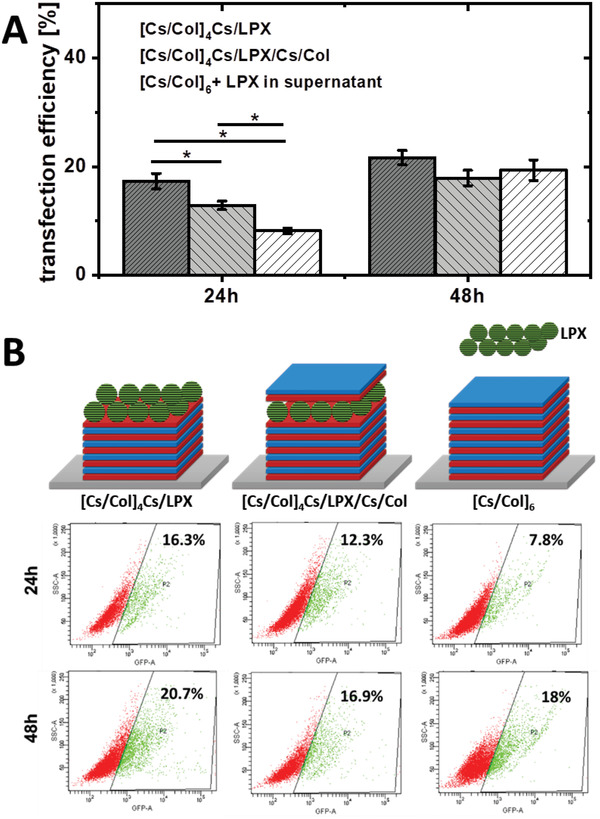
Transfection efficiency of the three examined PEMs sequences: [Cs/Col]_4_Cs/LPX, [Cs/Col]_4_Cs/LPX/Cs/Col, and [Cs/Col]_6_ (LPX in supernatant). A) Transfection efficiency as fraction of GFP positive cells at 24 and 48 h after beginning of cell incubation. Error bars represent means ± SD (*n* = 3), statistically significant differences using one‐way ANOVA followed by Scheffé post hoc test, *α* = 0.05, with a *p*‐value ≤ 0.05. B) Representative flow cytometry dot plots representing sight scatter of cell light scattering (SSC) and the fluorescence intensity in the GFP‐sensitive channel (GFP‐A) of hADSCs seeded on [Cs/Col]_6_ (LPX in supernatant), [Cs/Col]_4_Cs/LPX, and [Cs/Col]_4_Cs/LPX/Cs/Col. Green color corresponds to GFP positive cells with a higher fluorescence signal compared to the auto fluorescence of cells, and red color to the GFP‐ negative cells. The negative control of cells growing on [Cs/Col]_6_ in the absence of LPX is presented in Figure S6, Supporting Information.

The DNA transfer activity of the gene‐activated PEM was evaluated using a pDNA encoding for GFP as reporter gene (**Figure** **10**). Besides the DNA‐activated PEM [Cs/Col]_4_Cs/LPX/Cs/Col, the intermediate PEM [Cs/Col]_4_Cs/LPX (surface adsorbed LPX which were not protected by a cover layer) and the LPX‐free reference PEM [Cs/Col]_6_ with LPX in the supernatant, were used for comparison. These controls enable an assessment of the influence of the cover layer on the transfection on the one hand and whether embedding LPX in PEMs has an impact on the transfection efficiency on the other. After 24 h, the highest efficiency was detected for the system [Cs/Col]_4_Cs/LPX (≈16% GFP positive cells), while the final PEM [Cs/Col]_4_Cs/LPX/Cs/Col showed a slightly reduced efficiency (≈12% GFP positive cells). The control with LPX in the supernatant showed the lowest intensity. At 48 h after incubation, no significant differences were observed among the three groups and efficiency values of ≈17–20% GFP positive hADSCs were detected. The immobilization of LPX seems to trigger a fast contact between LPX and cells, leading to higher efficiency of the systems with PEM bound LPX after 24 h. This kinetic effect levels off after 48 h. The results demonstrated that LPX immobilization had no diminishing effect on the efficacy of the LPX formulation in the time frame studied. In addition, the transfection efficacy of 20% was promising to proceed experiments with BMP‐2 encoding DNA as autocrine and paracrine effects could be expected when 20% of the hADSCs growing on the PEMs express the gene of interest.^[^ ^81^ ^]^

#### 3.4.3 Osteogenic Differentiation of Mesenchymal Stem Cells on BMP‐2‐Gene Activated LPX‐Loaded PEMs

**Figure 11:**
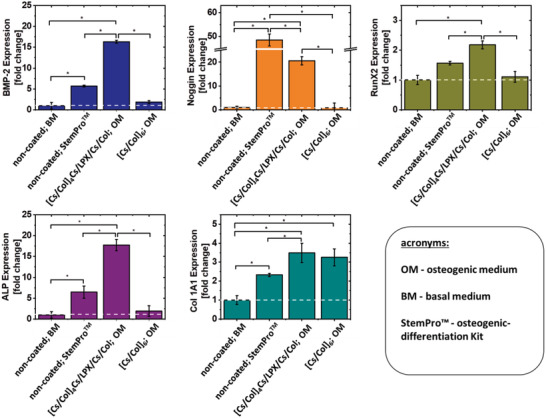
RT‐qPCR analysis of representative osteogenic markers: BMP‐2, ALP, Col1A1, Noggin, and RunX2. The results represent the gene expression at day 28 of the osteogenic differentiation experiments. For the BMP‐2 gene activated PEMs [Cs/Col]_4_Cs/LPX/Cs/Col hADSCs were seeded on the multilayer in OM, containing *β*‐Gly, Dex, and ASC but no BMP‐2 or other cytokines). Multilayer film control without LPX hADSCs was grown on [Cs/Col]_6_ in OM as medium to check for differentiation triggered by the two ECM components. For positive control, cells grew on non‐coated wells and were treated with StemPro osteogenic‐differentiation Kit from Thermo Fisher Scientific, containing supplements and cytokines for efficient osteogenic differentiation of hADSCs (non‐coated; StemPro). For negative control, hADSCs grew on non‐coated wells and were treated with BM (non‐coated; BM). Results represent means ± SD, with *n* = 3; statistically significant differences using one‐way ANOVA followed by Scheffé post hoc test, *α* = 0.05, with a *p*‐value ≤ 0.05.

In this section, the transfection‐active surface coating [Cs/Col]_4_Cs/LPX/Cs/Col was loaded with LPX with complex BMP‐2‐encoding DNA. Potentially, successful BMP‐2 expression can lead to autocrine or paracrine BMP‐2 effects by transfected cells. Gene expression analysis of specific osteoblast markers was performed by mRNA quantification. The expression of five osteogenesis‐related genes (RunX‐2, BMP‐2, ALP, Col1A1 and Noggin) was quantified by RT‐qPCR 28 days after hADSCs were seeded on [Cs/Col]_4_Cs/LPX/Cs/Col (**Figure** **11**). As medium for the experiments, we have chosen OM, which contained essential components for osteogenic differentiation: *β*‐Gly serves as a source of phosphate in hydroxyapatite structures, Dex has an enhancing stimulus on BMP‐2 effect, and ASC is an enhancer of collagen type 1 secretion.^[^ ^82^, ^83^ ^]^ The following controls where chosen: cells cultured in BM on surfaces without PEM coating were used as negative control due to the absence of inductors for osteogenic differentiation (Figure 11, non‐coated; BM). As positive control, hADSCs were cultured on surfaces without PEM coating using the StemPro Osteogenic‐Differentiation Kit provided by the supplier of the stem cells (Figure 11, non‐coated; StemPro). StemPro has an optimized mix of supplements and cytokines to reliably induce differentiation to osteocytes. As further necessary control, hADSCs in OM growing on [Cs/Col]_6_ in the absence of LPX were examined because of two reasons: 1) we know from our previous work that PEMs composed of ECM components can also trigger differentiation and want to estimate that effect^[^ ^53^, ^54^, ^57^ ^]^ and 2) due to the possibility to trigger osteogenic differentiation by Dex of the OM.^[^ ^84^ ^]^ Nevertheless, as biogenic glucocorticoids exert profound effects on bone and are essential for human osteoblast differentiation, we decided to keep Dex in the OM. The presence of BMP‐2 in Dex containing OM has enhancing effects on the osteogenic differentiation of stem cells.^[^ ^85^ ^]^

Analysis of the expression level of the mentioned osteogenic markers demonstrated that cells grown on BMP‐2 gene‐activated [Cs/Col]_4_Cs/LPX/Cs/Col exhibited enhanced expression of all osteo‐specific genes compared to the negative control (Figure 11). For BMP‐2, [Cs/Col]_4_Cs/LPX/Cs/Col showed a clearly increased expression by 15‐fold. This observation demonstrated the successful transfection and expression of the encoded gene in the DNA‐activated PEMs. The positive control (non‐coated; StemPro) also showed a 5‐fold increased BMP‐2 expression but significantly lower compared to the [Cs/Col]_4_Cs/LPX/Cs/Col multilayer. Osteogenic differentiation can also be associated with increased BMP‐2 expression, which accelerates the differentiation process.^[^ ^86^ ^]^ The LPX‐free PEM control film ([Cs/Col]_6_; OM) showed no significant difference to the negative control (non‐coated; BM) for the BMP‐2 expression. As an antagonist of BMP‐2, we investigated the expression of the osteogenic marker Noggin.^[^ ^87^ ^]^ Noggin was significantly upregulated for the positive control (non‐coated; StemPro), and the transfection system [Cs/Col]_4_Cs/LPX/Cs/Col also showed an increased expression of this marker (Figure 11). This was significantly higher for the positive control; although, the BMP‐2 expression was significantly lower compared to the BMP‐2 gene‐activated PEM. It is known from literature that Noggin has a biphasic dose‐dependent expression, and it has been reported that at lower BMP‐2 concentrations (0.01 to 1 µg mL^−1^), Noggin induction is enhanced. By contrast, induction of Noggin is diminished when BMP‐2 concentrations are shifted from 1 to 50 µg mL^−1^.^[^ ^88^ ^]^ This behavior could explain why the positive control has a lower BMP‐2 expression but more than twice as much Noggin expression as cells growing on the [Cs/Col]_4_Cs/LPX/Cs/Col multilayer. Nevertheless, this explanation remains speculative because the BMP‐2 amount in our experiments was not quantified, a focus of ongoing research. The third marker, RunX2, is one of the most important transcription factors, which is especially important in the early phase of osteogenic differentiation as it is upregulated in pre‐osteoblasts and downregulated in mature osteoblasts.^[^ ^89^ ^]^ For the sample [Cs/Col]_4_Cs/LPX/Cs/Col and the positive control, RunX2 is upregulated. The BMP‐2 gene‐activated PEM has significantly higher RunX2 values compared to all controls (Figure 11). The same expression pattern is found for ALP (Figure 11), which is needed to generate phosphate ions from natural sources for the hydroxyapatite matrix of bone tissues. The significantly highest ALP expression is found in cells growing on the [Cs/Col]_4_Cs/LPX/Cs/Col. The last screened marker was Col1A1 because expression of the Col1A1 gene occurs mainly during the shift from early to mature stages of osteoblast maturation, when the osteoblasts start building the ECM.^[^ ^90^ ^]^ The [Cs/Col]_4_Cs/LPX/Cs/Col, the positive control (non‐coated; StemPro), and the LPX‐free PEMs reference ([Cs/Col]_6_; OM) show a significantly increased Col1A1 expression compared to the negative control (non‐coated; BM) (Figure 11). That result can be expected for the [Cs/Col]_4_Cs/LPX/Cs/Col and the positive control because the other osteogenic markers are also increased in the expression analysis. For the LPX‐free [Cs/Col]_6_ reference, Col1A1 is the only marker screened in this study which has statistically significant increase compared to the negative control (non‐coated; BM). A closer look to the results for BMP‐2, RunX2, and ALP expression of cells on LPX‐free PEMs ([Cs/Col]_6_; OM) shows a slight but non‐significant increase of gene expression compared to the negative control (non‐coated; BM). It is known from previous research that Col/Cs PEMs can promote osteogenic differentiation,^[^ ^57^ ^]^ which was why we have chosen these composites as main component for the DNA‐activated PEM. Nevertheless, these studies demonstrated the additive effect of the ECM mimicking PEMs in combination with the in situ BMP‐2 transfection.

**Figure 12:**
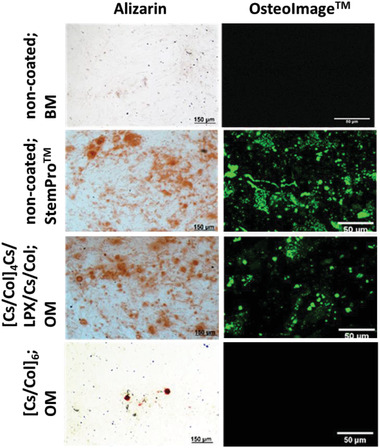
Histochemical (Alizarin) and fluorescence (OsteoImage) staining for inorganic bone matrix at day 24 of the differentiation experiment. The alizarin staining appears for calcium structures red, while OsteoImage fluorescently stains hydroxyapatite with high specificity for CLSM investigation (here shown in green). To prove osteogenic differentiation with the developed PEMs, hADSCs were seeded on BMP‐2 gene‐activated [Cs/Col]_4_Cs/LPX/Cs/Col in OM. PEMs without LPX was investigated with cells growing on [Cs/Col]_6_ in OM. For positive control, cells grew on non‐coated wells using the StemPro osteogenic‐differentiation Kit to induce osteogenic differentiation. For negative control, hADSCs grew on non‐coated wells using BM. Micrographs were taken for Alizarin staining at 10× magnification (bar represents 150 µm) and for OsteoImage staining at 40× magnification (bar represents 50 µm).

To further evaluate osteogenic differentiation, all samples were tested for mineralization (**Figure** **12**). At the final stage of osteoblast differentiation, the formation of mineralized nodules was a crucial phenomenon that indicates the maturation of osteoblasts. To evaluate this, Alizarin red staining was used to screen the degree of mineralization by visualizing calcium nodules after 24 days (stained red spots). Cells cultured on BMP‐2 gene activated [Cs/Col]_4_Cs/LPX/Cs/Col and positive control (non‐coated, StemPro) developed large Alizarin positive nodules, a strong indication of hADSC undergoing osteogenesis. The poor performance of mineral nodules for hADSCs growing on [Cs/Col]_6_ in OM may result from the lack of BMP‐2, again demonstrating the enhancing effect of in situ BMP‐2 transfection. As the bone ECMcontains hydroxyapatite, a phosphate mineral with the composition Ca_10_(PO_4_)_6_(OH)_2_, a method for a mineral‐specific staining provides more information than non‐specific Alizarin staining. OsteoImage^TM^, a commercially available hydroxyapatite specific fluorescence dye, was used to screen for these osteo‐specific mineral deposition. The images from this staining confirm the results from alizarin red staining (Figure 12). Both the positive control (non‐coated, StemPro) and the BMP‐2 gene activated [Cs/Col]_4_Cs/LPX/Cs/Col show pronounced hydroxyapatite structures after 24 days, which are absent in the negative control (non‐coated, BM) and for hADSCs on [Cs/Col]_6_ in OM. Concluding, the data from RT‐qPCR are supported by these staining for the inorganic ECM components of bone tissue. The BMP‐2 gene‐activated PEM can induce osteogenic differentiation of hADSCs comparable to the positive control.

## 4 Conclusion

The main goal of this work was to develop a novel gene‐functionalized ECM‐mimicking multilayered thin film for implants coating. We demonstrated that it is possible to entrap LPX into PEMs composed of the bone ECM components Cs and Col to engineer a nanoparticle functionalized thin film surface coating. The affinity of stem cells to the surface coating was proven as well as its ability of contact triggered transfection of cells growing on the gene‐activated PEMs, triggering their differentiation into the osteogenic lineage. The transfection activity allows an in situ cytokine production which is spatially and temporally restricted due to the contact triggered transfection of cells with a non‐viral gene delivery system, which only allows episomal gene uptake in cells resulting in a transient genetic modification. Hence, this PEM system is promising for clinical application as implant coating for bone tissue regeneration due to its camouflaging effect by mimicking bone ECM, providing an effective biological niche for osteogenic cell differentiation. The presented PEM‐LPX system can also be used as an mRNA delivery system because mRNA also allows transient protein expression with promising opportunities for regenerative medicine.^[^ ^91^ ^]^ Therefore, this system is of high interest to develop novel alternatives for implant coating for future in vivo use in bone tissue regeneration and in other tissue engineering applications.

## Conflict of Interest

The authors declare no conflict of interest.

## Supporting information

Supporting Information

## Data Availability

Research data are not shared.
