# Supplementary material for: Lipoplex‐Functionalized Thin‐Film Surface Coating Based on Extracellular Matrix Components as Local Gene Delivery System to Control Osteogenic Stem Cell Differentiation
Source: Adv Healthc Mater. 2022 Nov 29;12(5):2201978. doi: 10.1002/adhm.202201978 (PMC11469139; doi:10.1002/adhm.202201978)
Supplement: Supplementary file 1 — Supporting Information [file ADHM-12-2201978-s001.pdf]

ADVANCED HEALTHCARE MATERIALS

Supporting Information

for *Adv. Healthcare Mater.*, DOI 10.1002/adhm.202201978

Lipoplex-Functionalized Thin-Film Surface Coating Based on Extracellular Matrix Components as Local Gene Delivery System to Control Osteogenic Stem Cell Differentiation

Catharina Husteden, Yazmin A. Brito Barrera, Sophia Tegtmeyer, João Borges, Julia Giselbrecht, Matthias Menzel, Andreas Langner, João F. Mano, Christian E. H. Schmelzer, Christian Wölk and Thomas Groth**

Supplemental information

Lipoplex-functionalized thin-film surface coating based on extracellular matrix components as local gene delivery system to control osteogenic stem cell differentiation

Catharina Husteden[a]‡, Yazmin A. Brito Barrera[b]‡, Sophia Tegtmeyer[a], João Borges[c], Julia Giselbrecht[a], Matthias Menzel[d], Andreas Langner[a], João F. Mano,[c] Christian E.H. Schmelzer[d], Christian Wölk[e*], Thomas Groth[b,f*]

a) Martin Luther University Halle-Wittenberg, Institute of Pharmacy, Department of Medicinal Chemistry, Wolfgang-Langenbeck-Str. 4, 06120 Halle (Saale), Germany

b) Martin Luther University Halle-Wittenberg, Institute of Pharmacy, Department Biomedical Materials, Heinrich-Damerow-Str. 4, 06120 Halle (Saale), Germany

c) Department of Chemistry, CICECO - Aveiro Institute of Materials, University of Aveiro, Campus Universitário de Santiago, 3810-193 Aveiro, Portugal

d) Fraunhofer Institute for Microstructure of Materials and Systems (IMWS), Department of Biological and Macromolecular Materials, Walter-Hülse-Str. 1, 06120 Halle (Saale), Germany

e) Institute of Pharmacy, Pharmaceutical Technology, Faculty of Medicine, Leipzig University, 04317 Leipzig, Germany

f) Martin-Luther-University Halle-Wittenberg, Interdisciplinary Center of Materials Science, Heinrich-Damerow-Str. 4, 06120 Halle (Saale), Germany

‡ These authors contributed equally to this work.

corresponding author: Thomas Groth & Christian Wölk

*thomas.groth@pharmazie.uni-halle.de
*christian.woelk@medizin.uni-leipzig.de

Schematic illustration and selected images of gel electrophoresis experiment for the determination of the DNA loading efficiency after embedding LPX into PEM using an indirect method of quantifying the fraction of non-adsorbed DNA.

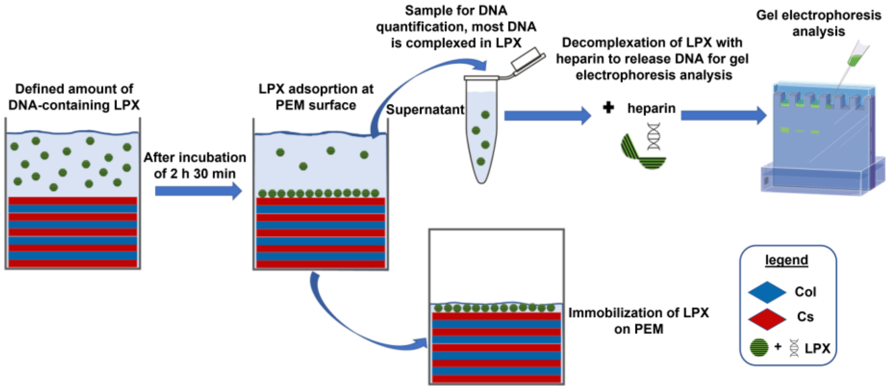

Figure S1. Schematic illustration of the indirect quantification of DNA loading of PEMs.

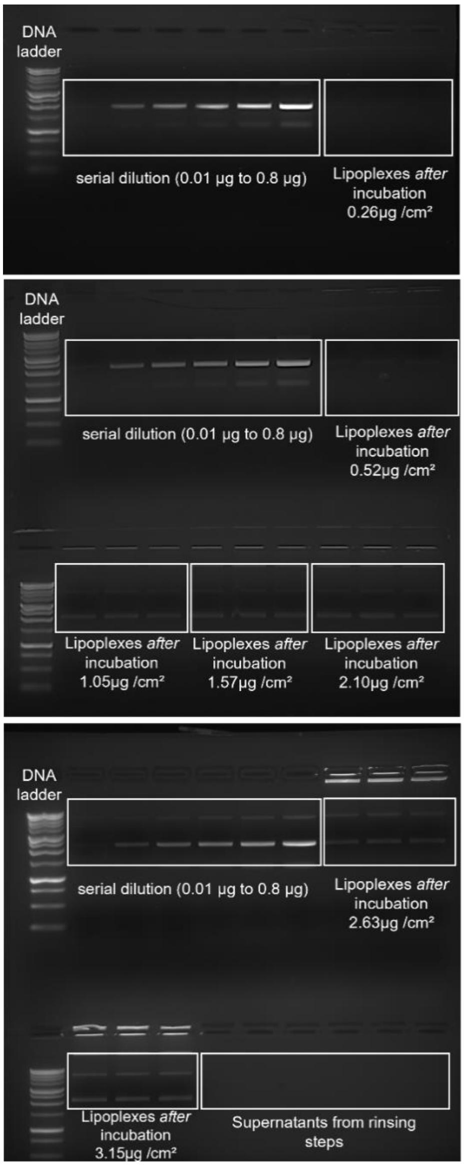

Figure S2. Representative agarose gel for the determination of the DNA loading of $[Cs/Col]_4Cs/LPX/Cs/Col$ films after incubation with different amounts of DNA encapsulated in LPX. The supernatant of the incubation solution was loaded on the gel to determine the adsorbed amount of DNA indirectly.

Atomic force microscopy

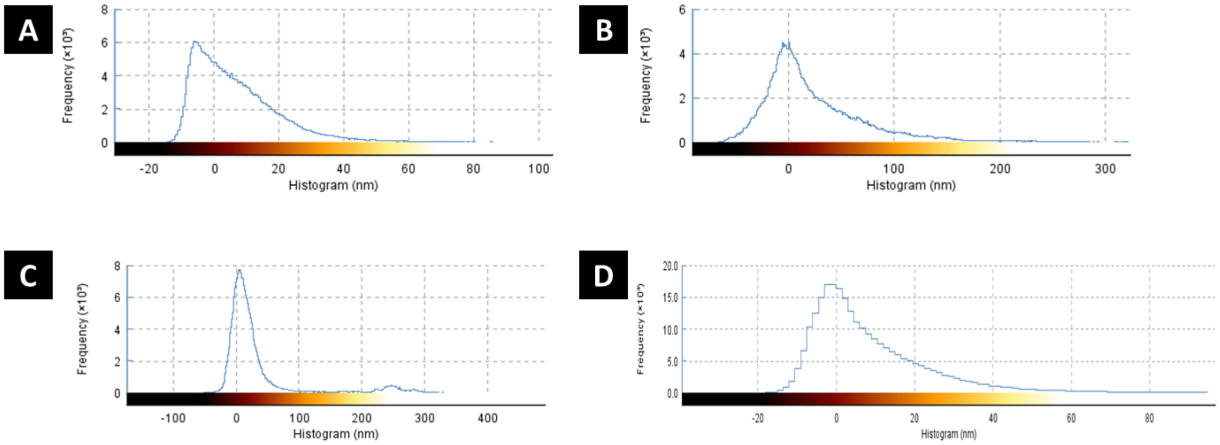

Figure S3. Figure 4 A-D) Histograms of height mode **(A)** [Cs/Col]$_4$Cs, **(B)** [Cs/Col]$_4$Cs/LPX, **(C)** [Cs/Col]$_4$Cs/LPX/Cs, and **(D)** [Cs/Col]$_4$Cs/LPX/Cs/Col by AFM.

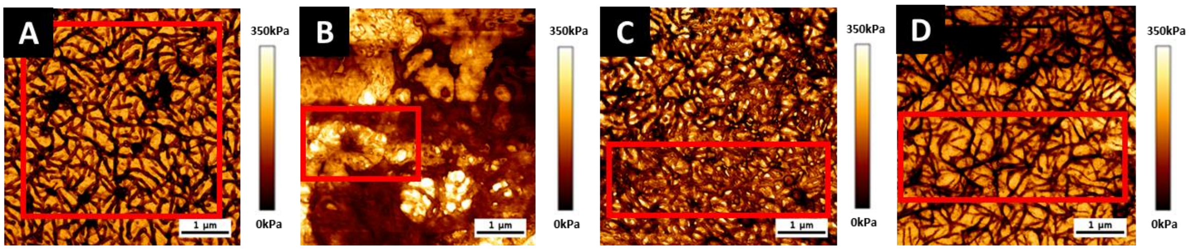

Figure S4. Stiffness micrographs of different preparation steps of the PEM system. **(A)** [Cs/Col]$_4$Cs, **(B)** [Cs/Col]$_4$Cs/LPX, **(C)** [Cs/Col]$_4$Cs/LPX/Cs, and **(D)** [Cs/Col]$_4$Cs/LPX/Cs/Col by AFM. Red square represents the selected area for the determination of the E_0 modulus. The reduced area of PEMs bearing LPX was necessary due to the inhomogeneity of the PEMs.

Table S1. Average third highest peak to third lowest valley height (R3Z ISO), waviness average (Wa) and root mean square waviness (Wq) distribution of PEM sequences before and after lipoplexes deposition. 1-dimensional roughness analysis, according to ISO 4287, 4288, 3274, mean values calculated from 10 separate lines, ln= 5µm, Dc= 1µm, cutoff filter: 0.02 measured by AFM

	R3z ISO [nm]	W_a [nm]	W_q [nm]
**[Cs/Col]$_4$Cs**	28.5 ±4.7	4.0 ±1.2	5.2 ±1.4
**[Cs/Col]$_4$Cs/LPX**	59.1 ±12.6	31.1 ±8.5	37.3 ±10.6
**[Cs/Col]$_4$Cs/LPX[Cs]**	42.3 ±4.9	7.3 ±2.5	9.4 ±3.9
**[Cs/Col]$_4$Cs/LPX[Cs/Col]**	35.7 ±9.1	5.8 ±1.2	7.2 ±1.5

Uptake studies of LPX in hADSCs

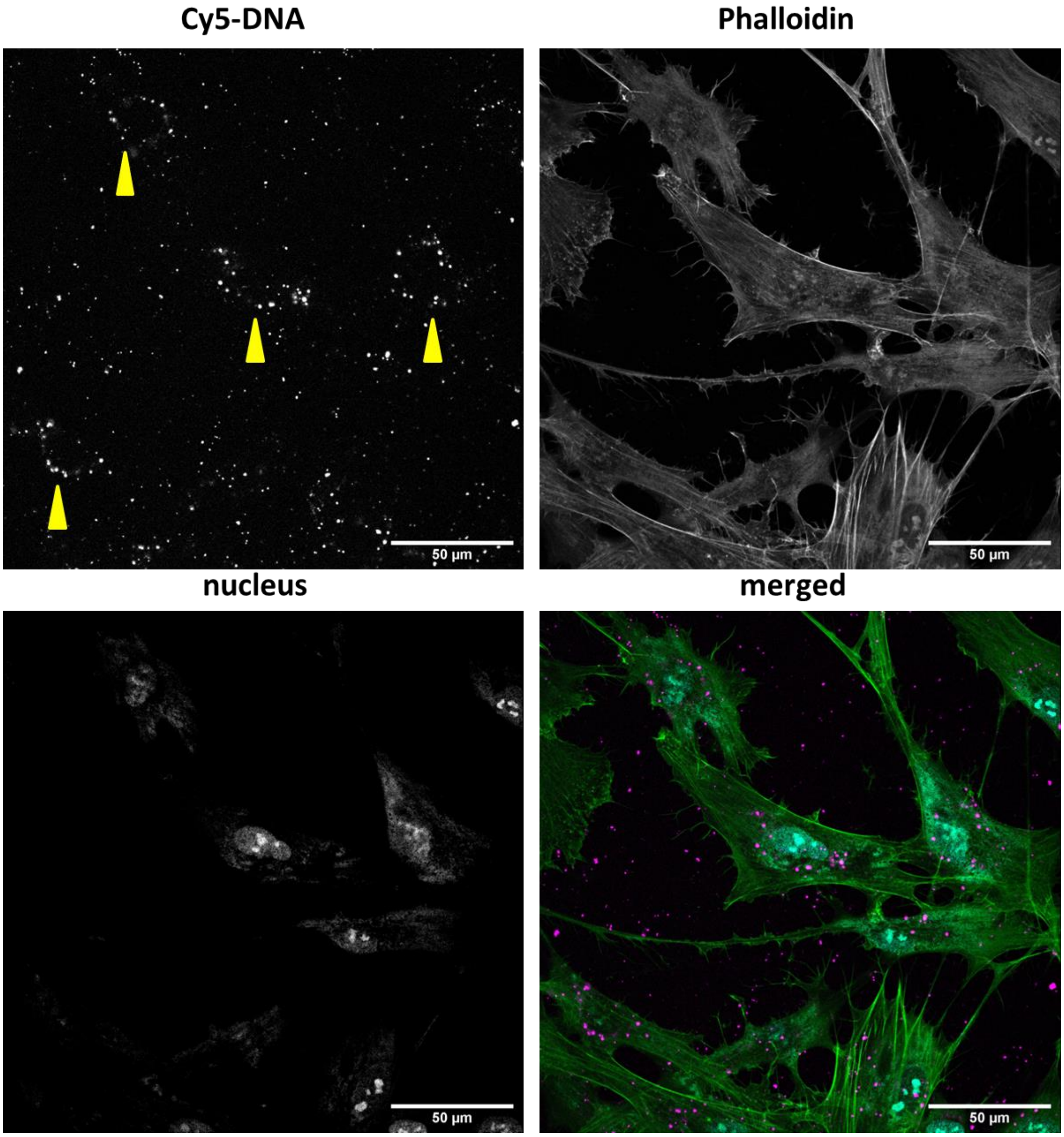

Figure S5. CLSM micrograph of transfected hADSCs after 48 h growing on $[Cs/Col]_4$Cs/LPX/Cs/Col with Cy-5 LabelIT® labelled DNA (merged image magenta). The cells are stained for filamentous actin with Phalloidin-Atto 488 (merged image green) and nuclei with BOBO-1 (merged image cyan). Images were taken at 40x magnification and evaluated with ImageJ. Images are given as single channels and merged. The given image is an optical slight of the basal part taken by CLSM analyses. The bar represents 50 µm.

Transfection studies with flow cytometry

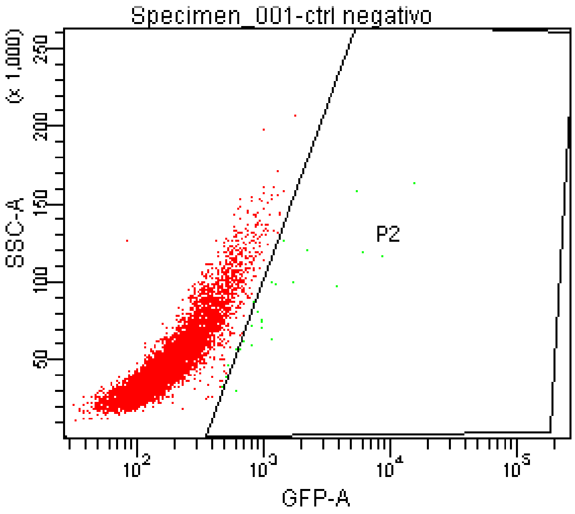

Figure S6. Flow cytometry dot plots representing sight scatter of cell light scattering (SSC) and the fluorescence intensity in the GFP-sensitive channel (GFP-A) of hADSC cells seeded on $[Cs/Col]_6$ in absence of LPX as negative control of the transfection studies.
